# AUXIN RESPONSE FACTOR 2 Intersects Hormonal Signals in the Regulation of Tomato Fruit Ripening

**DOI:** 10.1371/journal.pgen.1005903

**Published:** 2016-03-09

**Authors:** Dario A. Breitel, Louise Chappell-Maor, Sagit Meir, Irina Panizel, Clara Pons Puig, Yanwei Hao, Tamar Yifhar, Hagai Yasuor, Mohamed Zouine, Mondher Bouzayen, Antonio Granell Richart, Ilana Rogachev, Asaph Aharoni

**Affiliations:** 1 Department of Plant and Environmental Sciences, Weizmann Institute of Science, Rehovot, Israel; 2 Instituto de Biología Molecular y Cellular de Plantas, Consejo Superior de Investigaciones Cientificas- Universidad Politécnica de Valencia, Valencia, Spain; 3 University of Toulouse, INPT, Laboratory of Genomics and Biotechnology of Fruit, Castanet-Tolosan, France; 4 INRA, UMR990 Génomique et Biotechnologie des Fruits, Chemin de Borde Rouge, Castanet-Tolosan, France; 5 Plant Stress Physiology Lab, Gilat Research Center, Agricultural Research Organization (ARO), Rural Delivery Negev, Israel; The University of North Carolina at Chapel Hill, UNITED STATES

## Abstract

The involvement of ethylene in fruit ripening is well documented, though knowledge regarding the crosstalk between ethylene and other hormones in ripening is lacking. We discovered that AUXIN RESPONSE FACTOR 2A (ARF2A), a recognized auxin signaling component, functions in the control of ripening. *ARF2A* expression is ripening regulated and reduced in the *rin*, *nor* and *nr* ripening mutants. It is also responsive to exogenous application of ethylene, auxin and abscisic acid (ABA). Over-expressing *ARF2A* in tomato resulted in blotchy ripening in which certain fruit regions turn red and possess accelerated ripening. *ARF2A* over-expressing fruit displayed early ethylene emission and ethylene signaling inhibition delayed their ripening phenotype, suggesting ethylene dependency. Both green and red fruit regions showed the induction of ethylene signaling components and master regulators of ripening. Comprehensive hormone profiling revealed that altered *ARF2A* expression in fruit significantly modified abscisates, cytokinins and salicylic acid while gibberellic acid and auxin metabolites were unaffected. Silencing of *ARF2A* further validated these observations as reducing *ARF2A* expression let to retarded fruit ripening, parthenocarpy and a disturbed hormonal profile. Finally, we show that ARF2A both homodimerizes and interacts with the ABA STRESS RIPENING (ASR1) protein, suggesting that ASR1 might be linking ABA and ethylene-dependent ripening. These results revealed that ARF2A interconnects signals of ethylene and additional hormones to co-ordinate the capacity of fruit tissue to initiate the complex ripening process.

## Author Summary

The hormone ethylene is known to be involved in fleshy fruit ripening, although the role of other hormones is less well studied. Here we investigated the role of AUXIN RESPONSE FACTOR 2A (ARF2A) in tomato fruit ripening and suggest that it may be involved in the crosstalk between ethylene and other hormones. We show that over-expression of *ARF2A* (*ARF2-OX*) causes the fruit to ripen in an uneven, blotchy manner. The timing of ripening in *ARF2-OX* fruit is affected by applying exogenous ethylene, but the variegated appearance of ripening regions is independent of ethylene. In agreement with findings in *ARF2-OX* fruit, silencing of both *ARF2* paralogs, *ARF2A* and *ARF2B* (*ARF2as*), delayed the ripening process. Comprehensive hormone profiling revealed that altered *ARF2* expression in fruit significantly impacted abscisates, cytokinins and salicylic acid while gibberellic acid and auxin metabolites were unaffected. Transcriptome analysis of *ARF2-OX* fruit patches revealed that normal ripening does occur, however, the timing and co-ordination is affected. These observations were reinforced in *ARF2as* fruit that displayed the opposite gene expression and metabolic phenotypes. Finally, we show that ARF2A homodimerizes as well as interacts with the known ABA STRESS RIPENING (ASR1) protein, suggesting that ASR1 might be linking ABA and ethylene-dependent ripening. Our results reveal that ARF2A may interconnect signals of ethylene and additional hormones to co-ordinate the capacity of fruit tissue to initiate the complex ripening process.

## Introduction

The significance of the gaseous hormone ethylene in the ripening of fleshy fruit has been recognized for almost sixty years [1]. Whether direct or not, it is now evident that ethylene influences the ripening of both climacteric and non-climacteric fruit [2, 3]. Though ethylene is considered to be the major hormonal regulator in climacteric fruit ripening, other hormones, such as auxin and abscisic acid (ABA), were shown to take part in this process [4, 5]. In non-climacteric fruit such as strawberry and grape, auxin and ABA, but not ethylene, are considered to play major roles in mediation of the ripening process [6, 7]. Hitherto, in fruit belonging to either class, our current knowledge regarding the crosstalk between ethylene and other hormones during ripening is very limited.

In tomato, mutants altered in pathways of hormone biosynthesis or signaling such as the auxin pathway mutant *diageotropica (dgt*) and the gibberellic acid (GA) pathway mutant *parthenocarpic fruit (pat*), provided evidence for the role of hormones other than ethylene in fruit development and ripening [8, 9]. These studies pointed towards the involvement of a multi-hormone signaling pathway in fruit ripening, for instance, tomato fruit with reduced ABA biosynthesis also displayed reduced ethylene emission [10]. Furthermore, exogenous ABA treatment was shown to promote various characteristics of the fruit ripening process [11]. The involvement of auxin in ripening was implicated by down-regulation of *ARF4* which resulted in dark green immature fruit, blotchy ripening and altered pectin structures [12, 13]. A direct effect with respect to fruit development and ripening was demonstrated in banana and tomato, where fruit treated with exogenous auxin displayed an increase in ethylene biosynthesis and accelerated ripening [14, 15]. The cross-regulation of auxin and ethylene was demonstrated in other organs in tomato as it was shown to have an effect on both root development [16] and in the process of abscission [17]. Finally, auxin movement was shown to be inhibited in transgenic tomato plants with reduced levels of the ethylene receptor *ETR1* [18].

The auxin response pathway in plants involves two major protein families, namely AUXIN RESPONSE FACTORS (ARFs) and AUXIN-INDUCED proteins (Aux/IAAs) [19, 20]. Members of the Aux/IAA family suppress expression of genes in the auxin signaling pathway by interfering with ARF activity. This is carried out via their carboxy-terminus dimerization domain which interacts with the dimerization domain in the ARF proteins in an auxin-dependent manner [21]. In addition to the carboxy-terminus dimerization domain, ARF proteins contain an amino-terminus DNA-binding domain which binds to auxin responsive elements (AuxREs) in promoter regions of auxin-responsive genes. The central region of ARF proteins indicates whether it will act as an activator or repressor of gene expression [22]. It is generally accepted that some ARF proteins affect transcription when bound to AuxREs as dimers but are inactive when bound to Aux/IAA proteins. Once auxin accumulates to significant levels, Aux/IAA proteins are directed to proteosomal degradation in a ubiquitin-dependent manner. Upon Aux/IAA degradation, ARFs are released and suggested to homo- or hetero-dimerize to regulate downstream gene expression [23]. Since there is a large number of Aux/IAA and ARF encoding genes, it was further suggested that a complex network of auxin response signaling exists and each or several ARF and Aux/IAA proteins regulate different processes involving a different set of auxin responsive genes [24]. In addition, *ARF* transcripts have been shown to be regulated by small RNAs, for example *ARF2*, *ARF3* and *ARF4* were shown to be cleaved by *trans-*acting short-interfering RNA (tasi-RNA) through sequence-specific recognition [25, 26].

The role of ARF2 in diverse developmental processes has been demonstrated previously. In Arabidopsis, the *arf2* mutant displayed large and dark green rosette leaves, late flowering, disturbed flower morphology, sterility and abnormal organ size such as larger seeds and elongated sepals [27, 28]. The ARF2 protein was also reported to be involved in vegetative phase change in maize and Arabidopsis in which *arf2* mutant plants exhibited delayed senescence in many parameters such as chlorophyll content, initiation of flowering, floral organ abscission and silique maturation [29, 30]. Moreover, it was suggested that in tomato and Arabidopsis, ARF2 activity is modulated by Aux/IAA3 and is associated with the auxin—ethylene crosstalk and apical hook formation [31, 32]. This suggests that ARF2 may stand at the core of a regulatory network bringing ethylene, auxin and light signaling together in these model plant species. A recent study revealed that two tomato ARF2 paralogs (i.e. ARF2A and ARF2B), are part of the ripening regulatory network [33]. Downregulation of both *ARF2* genes was shown to have a marked negative effect on tomato fruit ripening and expression of key ripening regulators was reduced.

In this study we provide complementary evidence that ARF2A plays a role in mediating the intricate hormonal interaction triggering the ripening process in fruit. Our findings were largely based on over-expression of *ARF2A* in tomato that resulted in a blotchy ripening pattern in fruit and accelerated ripening. The involvement of *ARF2A* in ethylene-dependent ripening is supported by its expression during fruit development, in the background of ripening mutants and in the earlier emission of ethylene in *ARF2A* over-expression fruit. In addition, exogenous application of both ethylene and auxin induced *ARF2A* expression and inhibition of ethylene receptors with 1-methylcyclopropene (1-MCP) reduced *ARF2A* levels. *ARF2A* transcripts in the fruit were also responsive to abscisic acid application. Multi-class hormone profiling revealed a significant impact of *ARF2A* over-expression and silencing on abscisates, cytokinins and salicylic acid while levels of GAs and auxin were unchanged. Additionally, protein interaction assays revealed that in addition to homo-dimerizing, ARF2A interacts with the known ripening-associated protein ABA STRESS RIPENING 1 (ASR1). Taken together, the results provide diverse lines of evidence that ARF2A interconnects signals of auxin, ethylene and likely additional hormone classes to prime the fruit tissue capacity to ripen by altering its sensitivity to ethylene.

## Results

### *ARF2A* expression is ripening-regulated in tomato fruit

**Fig 1:**
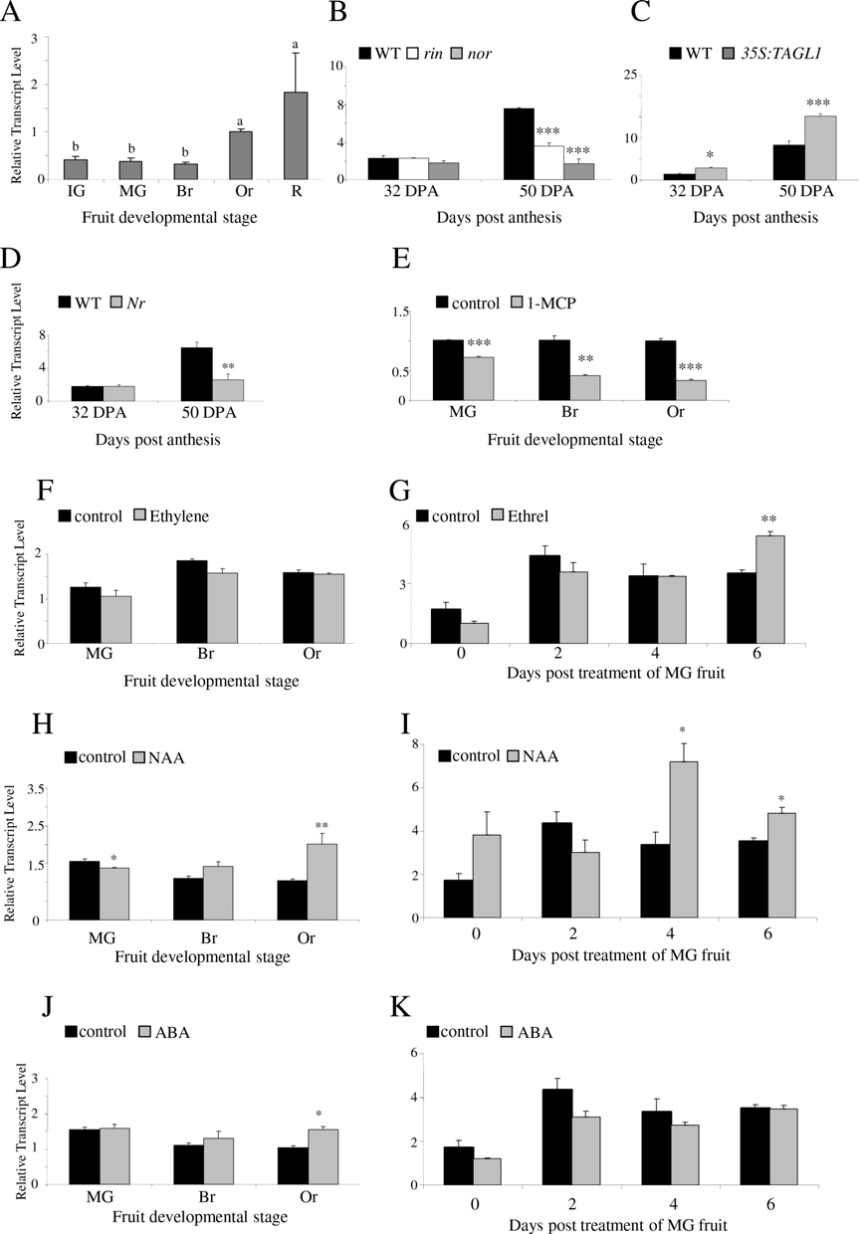
*ARF2A* expression in tomato fruit. Relative expression levels of *ARF2A* analyzed by qRT-PCR, in (A) WT fruit at five developmental stages; (B) *rin* and *nor* mutants; (C) *TAGL1* over-expressing fruit (*35S*:*TAGL1*); and (D) *nr* mutant. Relative expression levels of *ARF2A* in fruit at three developmental stages, treated with (E) 1-MCP; (F) ethylene; (H) NAA; and (J) ABA. Relative expression levels of *ARF2A* in fruit at the MG stage, at 0, 2, 4 and 6 days post-treatment with (G) ethrel; (I) NAA; and (K) ABA. Error bars represent SE. Statistical significance was evaluated using a student’s t-test, *p-value<0.05, **p-value<0.01 and ***p-value<0.001; dpa: days post anthesis; IG: immature green; MG: mature green; Br: breaker; Or: orange; and R: red.

To discover regulatory proteins associated with the shift to ripening we mined previously generated tomato transcriptome data for ripening-regulated genes [34]. The transcript of *AUXIN RESPONSE FACTOR 2* (*ARF2A*) displayed increased expression during ripening and we therefore selected it for deeper investigation. The expression level of *ARF2A* increased at the orange (Or) and red (R) fruit stages (Fig 1A). We next analyzed *ARF2A* transcript levels in the ripening impaired *ripening inhibitor (rin*) and *non-ripening (nor*) mutants and found that it was significantly reduced at 50 days post anthesis (dpa), a point in development where wild type (WT) fruit are red and ripening occurs (Fig 1B). However, no difference was observed between the WT and mutant fruit at an earlier time point, 32 dpa, a stage equivalent to the mature green (MG) stage in WT fruit. Four putative RIN-binding motifs [35] were identified at 956, 788, 623 and 498 base pairs upstream to the *ARF2A* start codon. *ARF2A* was recently reported to be a potential direct RIN target in ChIP-chip experiments [36], therefore reinforcing the possibility that *ARF2A* may be regulated by this ripening regulator. Moreover, its expression level was upregulated in both green and red stages of fruit over-expressing *TOMATO AGAMOUS-LIKE 1 (TAGL1*; Fig 1C), a MADS-box transcription factor reported to act as a positive regulator of tomato fruit ripening [37, 38]. The *ARF2A* expression profile, its down-regulation in the *rin* and *nor* ripening mutants, its direct binding by RIN and increase in the *TAGL1* over-expression lines associated *ARF2A* to the fruit ripening process in tomato.

### *ARF2A* expression responds to changes in ethylene levels and signaling as well as other hormonal cues

We used several mutants and treatments to examine the impact of ethylene and additional hormones on *ARF2A* expression levels in fruit. The tomato *Never-ripe (Nr*) mutant contains a mutation in the ethylene receptor ETR3 (NR). As in the *rin* and *nor* mutant fruit (Fig 1B), *ARF2A* expression was not altered at 32 dpa (*i*.*e*. MG fruit stage), but was significantly reduced in the *Nr* mutant at 50 dpa fruit (R stage) (Fig 1D). Fruit treated with the ethylene receptor inhibitor 1-methylcyclopropene (1-MCP) exhibited a significant reduction in *ARF2A* transcript levels when treated at the MG, breaker (Br) and orange (Or) stages (Fig 1E). Nevertheless, ethylene treatment of fruit at the same developmental stages did not have an effect on *ARF2A* transcripts (Fig 1F). In order to verify the efficacy of the ethylene treatment, the levels of *1-AMINO-CYCLOPROPANE-1-CARBOXYLIC ACID SYNTHASE4* (*ACS4*) expression were analysed (S1 Fig). Since *ACS4* expression is positively regulated by ethylene [39], a significant elevation in the expression levels of *ACS4* in the ethylene-treated MG and Br fruit indicated that the ethylene treatment was efficient. Hence, we can conclude that the lack of *ARF2A* response to the treatment was not due to experimental factors. We subsequently examined *ARF2A* expression in response to ethylene in a single developmental stage (MG) at several timepoints post-treatment. Application of the ethylene-releasing chemical ethrel [40], resulted in increased *ARF2A* transcript level only at six days post-treatment (Fig 1G). The relatively slow response of *ARF2A* expression to ethylene suggests that this response could be indirect, or might depend on other signals rather than merely exposure to ethylene.

The impact of abscisic acid (ABA) and 1-naphthaleneacetic acid (NAA) on *ARF2A* expression was also examined by treating fruit at the MG, Br and Or stages with these hormones and measuring *ARF2A* levels at three days post-treatment. An increase in *ARF2A* expression levels was observed after treatment with both NAA and ABA at the Or stage (Fig 1H–1J). In addition, a reduction in *ARF2A* levels was seen after NAA treatment at the MG stage (Fig 1H). We subsequently measured *ARF2A* expression at later timepoints post-treatment of the MG stage fruit with NAA or ABA. While no significant change was observed in its transcript level up to six days post ABA treatment (Fig 1K), NAA induced upregulation of *ARF2A* at four to six days post-treatment (Fig 1I). The results suggest that an association between ethylene, auxin and ABA signaling and the control of *ARF2A* expression.

### Silencing of *ARF2* in tomato supports its involvement in the tomato fruit ripening process

**Fig 2:**
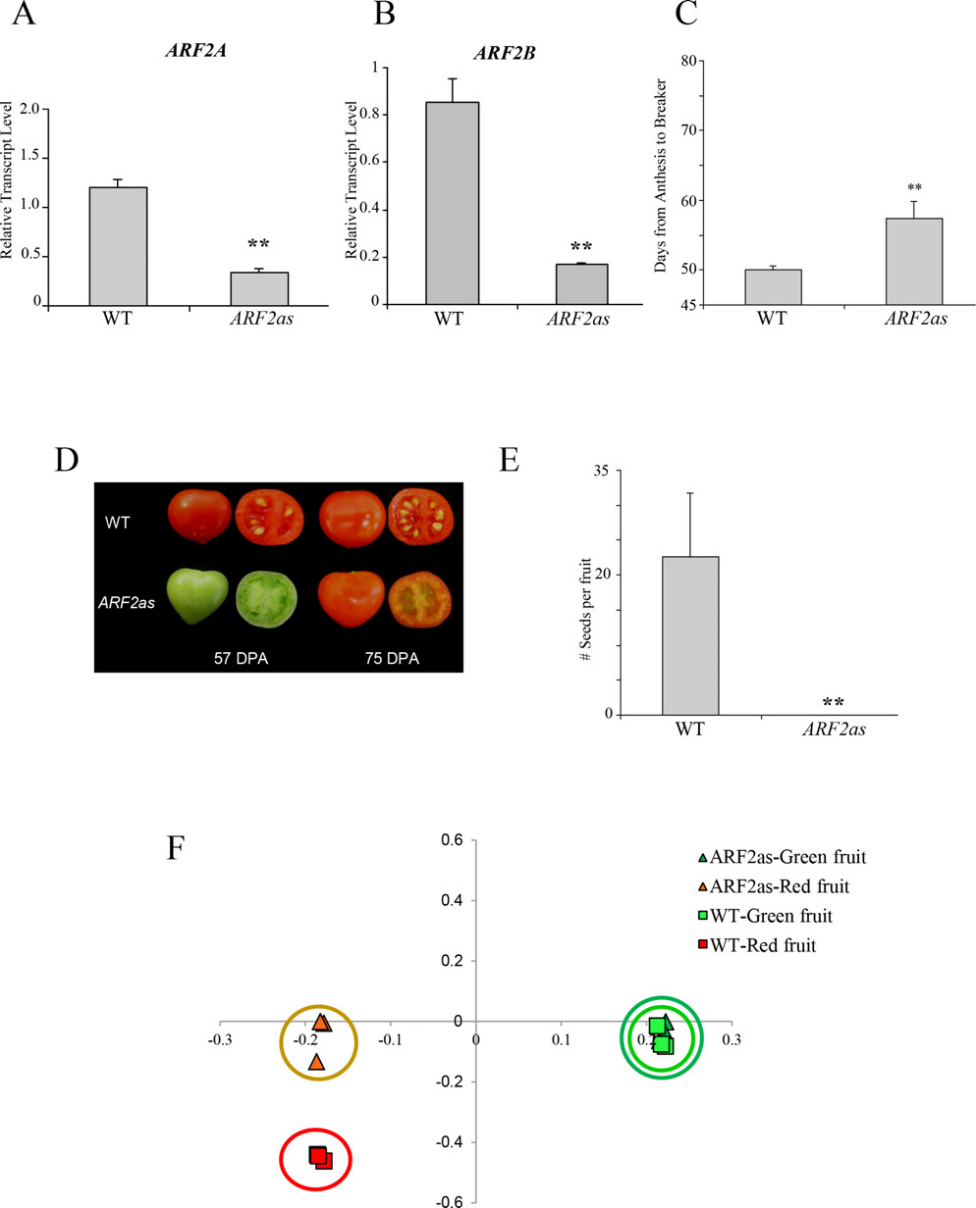
Phenotype and expression levels of *ARF2* genes in *ARF2as* transgenic lines. Fruit of *ARF2as* lines were analysed for relative expression levels by qRT-PCR of (A) *ARF2A;* and (B) *ARF2B*. (C-D) The ripening of *ARF2as* fruit is delayed as compared to WT. (D-E) *ARF2as* fruit were parthenocarpic or nearly parthenocarpic with reduced number of seeds. (F) Principle component analysis (PCA) plot from untargeted analysis of metabolites. DPA: days post anthesis; error bars represent SE; Statistical significance was evaluated using a student’s t-test, **p-value<0.01.

In order to further study the role of *ARF2* in tomato fruit ripening, we generated transgenic plants with reduced *ARF2* levels, using an antisense expression vector (*ARF2as*). The *ARF2as* construct was designed to downregulate both *ARF2A* as well as its close homolog *ARF2B* (S2 Fig). Expression analysis indicated that indeed both *ARF2A* and *ARF2B* were down-regulated in the red fruit of three of the seven *ARF2as* lines obtained (Figs 2A, 2B and S3). Fruit of the *ARF2as* lines exhibited delayed fruit ripening process, and the time from anthesis to breaker was significantly extended relative to WT fruit (Fig 2C). In addition, the fruit were either parthenocarpic or contained only a few seeds (Fig 2D and 2E).

A related study [33] performed in parallel to our study here investigated the role of *ARF2A* in tomato fruit ripening by down-regulating *ARF2A* and its homolog *ARF2B* using an RNAi approach. The authors describe a similar delayed ripening phenotype in their RNAi lines as we do here in with an antisense construct (Fig 2C and 2D). They conducted extensive gene expression analysis demonstrating reduced expression of many ripening-related genes. To complement this work, we examined the expression level of seven key ripening regulators in *ARF2as* fruit: *RIN*; *AP2a*; *NOR*; *TAGL1*; *NR*; *ETHYLENE RESPONSE FACTOR E1* (*ERF*.*E1*); and *COLORLESS NON-RIPENING* (*CNR*; S4 Fig). All of these ripening regulators were downregulated in the *ARF2as* fruit at the red stage. The gene expression data obtained correlated with the delayed-ripening phenotype observed in the *ARF2as* fruit.

### Fruit with reduced *ARF2* levels display altered ripening-associated metabolism

Metabolite analysis using high-resolution LC-MS was performed with extracts derived from WT and *ARF2as* fruit at the green and red stages. A PCA plot in which the entire LC-MS profiles of the different samples were represented was generated. While no separation was observed between the green tissues, the WT and the *ARF2as* fruit samples could be clearly separated from each other at the red stage (Fig 2F and S1 Table).

**Fig 3:**
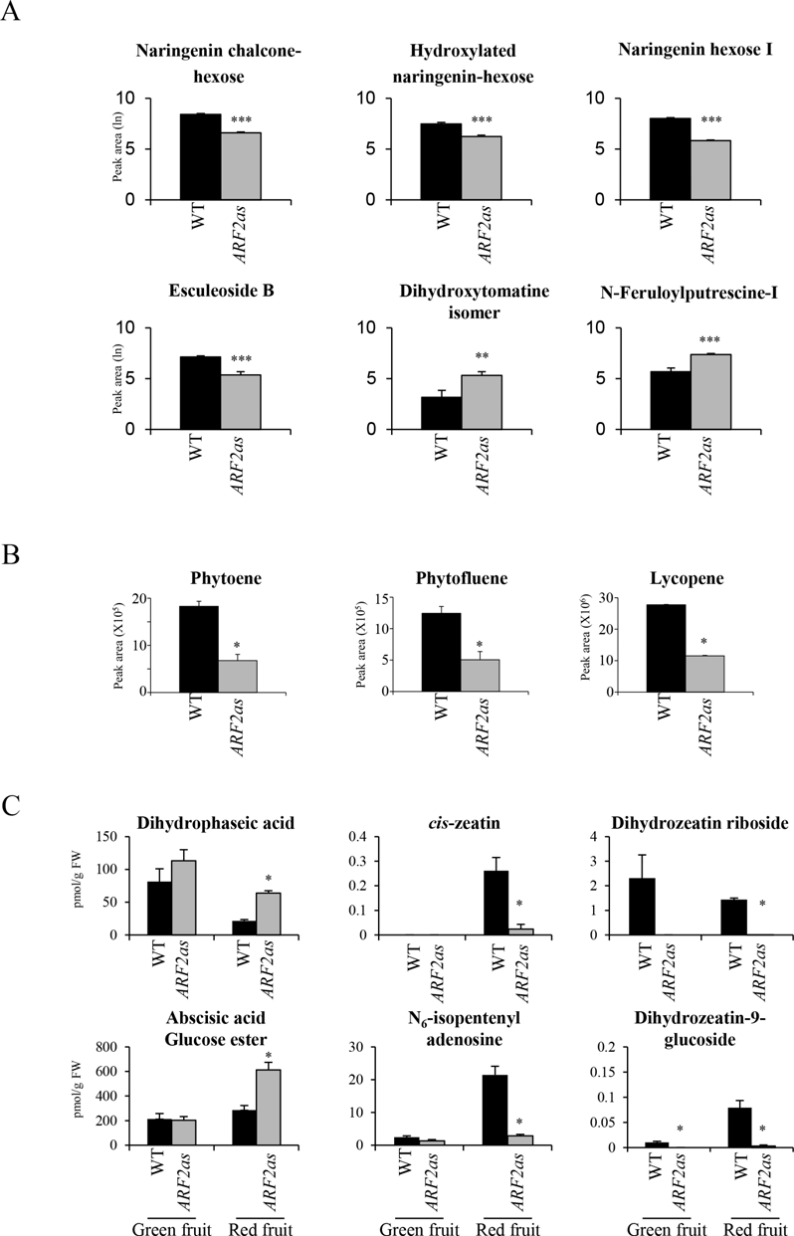
Metabolic analysis of *ARF2as* transgenic fruit. Accumulation in WT and *ARF2as* fruit of metabolites from the classes: (A) phenylpropanoids and glycoalkaloids (in red stage fruit); (B) isoprenoids (in red stage fruit, undetectable in green fruit); and (C) phytohormones (abscisates are presented in the two left graphs, and cytokinins in the middle and right panels; at both green and red stages of fruit ripening). WT samples are displayed as black bars and *ARF2as* as grey bars; error bars represent SE; statistical significance was evaluated using a student’s t-test, *p-value<0.05, **p-value<0.01, ***p-value<0.001; and DPA: days post anthesis.

We subsequently performed targeted analysis of fruit ripening associated secondary metabolites in the WT and *ARF2as* red fruit samples, mostly those belonging to the phenylpropanoid and steroidal glycoalkaloid classes. Nineteen putative metabolites were identified as having significantly different levels between the *ARF2as* and WT red fruit, in all of the three lines analysed (S2 Table), six of which are presented in Fig 3A. Ripening-associated secondary metabolite accumulation in *ARF2as* red fruit corresponded to their typical accumulation in a less advanced ripening stage than the WT red fruit. For example, seven naringenin derivatives from the phenylpropanoid pathway that were previously shown to accumulate upon ripening [41], accumulated to reduced levels in *ARF2as* fruit. Similarly, the ripening-associated glycoalkaloid, esculeoside B, exhibited reduced levels in *ARF2as* fruit (Fig 3A). Furthermore, compounds that are normally reduced upon ripening, such as the alkaloid dihydroxytomatine, chlorogenic acid and the polyamine N-feruloylputrescine, showed higher levels in the *ARF2as* fruit, in line with the inhibited ripening phenotype.

Employing a different analytical platform we also examined carotenoid and other isoprenoid levels in WT and *ARF2as* red fruit. Levels of phytoene, phytofluene and lycopene were all reduced in the red fruit of *ARF2as* as compared to WT (Fig 3B). Taken together, the results of altered ripening-related metabolite analysis and reduced gene expression of key ripening regulators are in line with the delayed ripening phenotype that *ARF2as* fruit possess.

We subsequently examined the hormonal profile in green and red fruit of the *ARF2as* fruit (Fig 3C and S3 Table). A total of 41 hormone metabolites belonging to five classes (CKs, IAAs, GAs, ABAs and SA) were examined. Both dihydrophaseic acid and abscisic acid glucose ester accumulated to higher levels in *ARF2as* red fruit (Fig 3C, leftmost panels). In addition, cytokinins were a major class of hormones altered in the *ARF2as* fruit. The levels of *cis*-zeatin and metabolites of the *trans*-cytokinins branch (dihydrozeatin riboside, dihydrozeatin-9-glucoside and N6-isopentenyl adenosine) were reduced in the red fruit of *ARF2as* (Fig 3C).

### Tomato fruit over-expressing *ARF2A* display accelerated and uneven ripening

**Fig 4:**
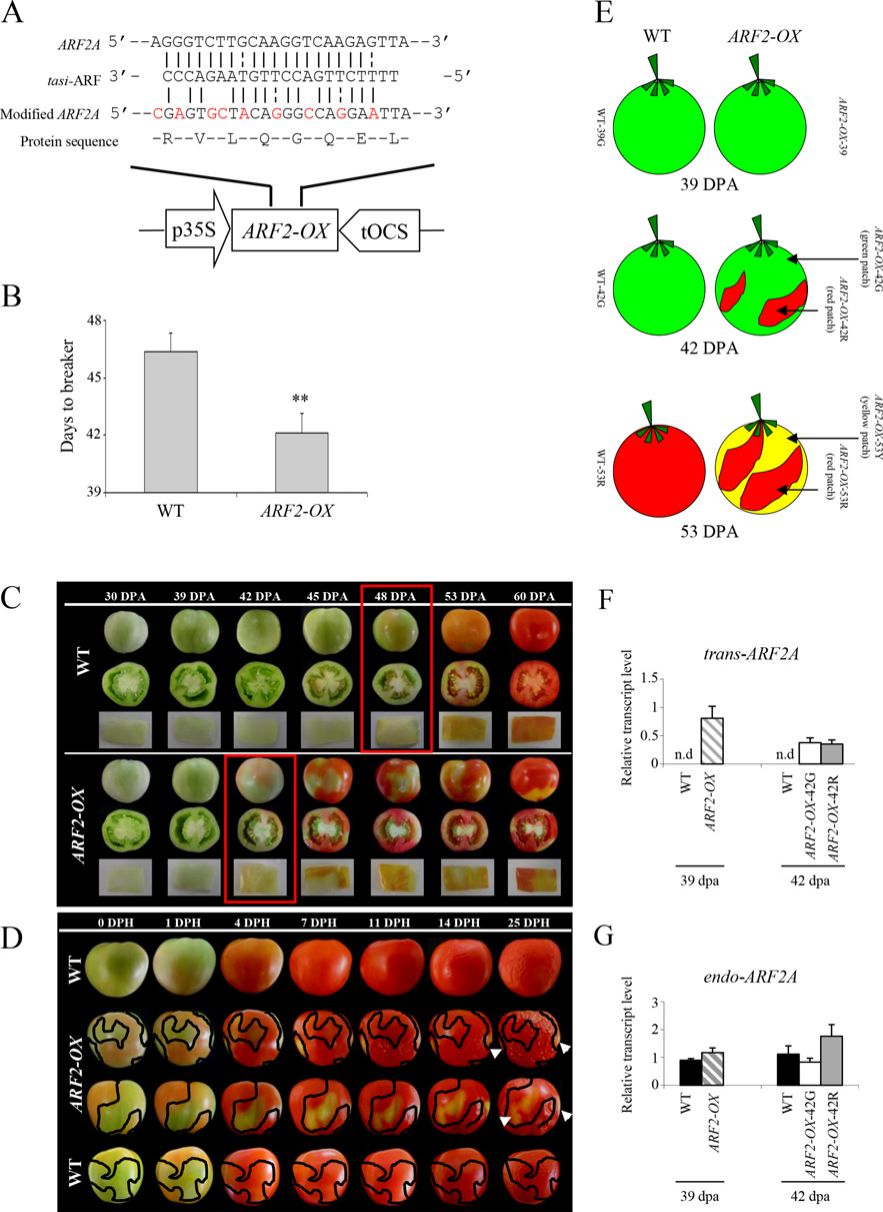
Over-expression of *ARF2A* in transgenic tomato plants. (A) Silent mutations in the tasi-RNA recognition site introduced into the *ARF2A* open reading frame; (B) days from anthesis to breaker stage in WT and *ARF2-OX* fruit (Error bars represent SE); (C) blotchy ripening phenotype observed in *ARF2-OX* fruit (DPA: days post anthesis); and (D) blotchy ripening occasionally observed in WT fruit which eventually all turn red in comparison with the *ARF2-OX* lines which have regions which remain yellow; (E) A scheme representing the sampling of tissues from WT and *ARF2-OX* fruit at 39, 42 and 53 dpa, in this study; when patches were visible at 42 and 53 dpa, they were harvested and treated separately. Relative expression levels of *ARF2A* variants were analyzed by qRT-PCR in WT and patches of *ARF2-OX* fruit at 39 and 42 dpa, using oligonucleotides specific to (F) the transgene (*trans-ARF2A*); and (G) the endogenous *ARF2A* gene (*endo-ARF2A*). Black bars represent WT, hatched bars *ARF2-OX* at 39 dpa, white bars *ARF2-OX* green patches at 42 dpa and grey bars *ARF2-OX* red patches at 42 dpa. Error bars represent SE. DPH: days post-harvest; n.d.: not detected; dpa: days post anthesis.

To examine other aspects of *ARF2A* and to further investigate the mechanism by which it is involved in fruit ripening together with its relation with hormone signaling, we generated tomato plants with constitutive *ARF2A* over-expression. The *ARF2A* coding sequence contains a *trans-*acting small interfering RNA (tasi-RNA) recognition site which may counteract over-expression of the gene (Fig 4A). Hence, to achieve robust over expression, we introduced silent mutations into the coding sequence to avoid tasi-RNA targeting while maintaining the amino acid sequence. Expression of the mutated *ARF2A* gene in tomato plants was driven by the CaMV 35S promoter and hereafter referred to as *ARF2-OX*. Eight independent transgenic lines were generated, five of which were evaluated for *ARF2A* gene expression. Three of these five were found to have upregulated expression levels of the *ARF2A* gene (S5 Fig). All three *ARF2-OX* lines with confirmed over-expression displayed a clear fruit phenotype (as described below; Fig 4B–4D). An additional three lines (out of the eight generated) were not evaluated for *ARF2A* expression, yet two of them displayed the same phenotype in fruit.

Fruit of *ARF2-OX* lines were markedly affected as they exhibited an accelerated ripening rate, reaching the Br stage an average six days prior to the WT fruit (Fig 4B). In addition, *ARF2-OX* fruit displayed an unusual, blotchy ripening pattern (Fig 4C); these patterns of fruit pigmentation were observed in both skin and flesh tissues; and in most cases the green regions turned red and became ripe while some failed to fully ripen and remained yellow. The yellow colour of the ripe regions resembled the inhibited ripening phenotype of known tomato ripening mutants, such as: *rin*, *nor* and *Nr* [42]. Unripe yellow regions stayed firm post-harvest and did not wrinkle with time, in contrast to the ripe, red regions (Fig 4D). The typical ripening of WT tomato fruit does not occur at once over the entire fruit but rather through a gradual, patchy process, as demonstrated by lycopene accumulation in selected regions during the Br to R stage transition (lower panel, Fig 4D). The phenotype observed in the *ARF2-OX* fruit was different as the regions were either green or red almost from the onset of ripening, with a clear margin. These results suggested that *ARF2A* could be involved in the co-ordination and regulation of signals that promote the process of fruit ripening.

### The *ARF2-OX* fruit ripening phenotype is a result of genuine over-expression of the *ARF2A* transgene

The blotchy ripening phenotype in the *ARF2-OX* fruit might have been a result of uneven expression of the *ARF2-OX* transgene. This could have occurred through either uneven silencing of the endogenous *ARF2A* in the form of variegated co-suppression or due to uneven over-expression of the *ARF2-OX* transgene by the 35S CaMV promoter driving it. To exclude these possibilities, and to demonstrate that the blotchy phenotype was a result of genuine *ARF2A* over-expression in an even manner over the entire fruit, we performed a detailed expression analysis of WT and *ARF2-OX* fruit. The following tissues were harvested and treated separately: *ARF2-OX* fruit at 39 dpa before patches are visible (*ARF2-OX*-39) and green and red patches from fruit at 42 dpa (*ARF2-OX*-42G and *ARF2-OX-*42R, respectively; Fig 4E). As control, WT fruit harvested at the same time points (39 and 42 dpa) were included. Transcript specific qRT-PCR assays were carried out with specific primers to independently analyze the endogenous *ARF2A* (*i*.*e*. *endo-ARF2A*) and the *ARF2-OX* transgene transcript (*i*.*e*. *trans-ARF2A*; Fig 4F and 4G). Analysis of *trans-ARF2A* expression showed that there was no significant difference between the various regions and across timepoints in the transgenic fruit (Fig 4F) and it was not detected in WT fruit, suggesting uniform over-expression. The *endo*-*ARF2A* transcript levels were not significantly different between the *ARF2-OX* and the corresponding WT fruit (Fig 4G). We also examined if other tomato *ARF* genes could have been variably co-suppressed in the *ARF2-OX* transgenic fruit. Of the four closest *ARF* family members, only the expression of *ARF4* was significantly downregulated in the *ARF2-OX* fruit, however, its expression was uniformly affected in both red and green patches (S6 Fig). In addition, the expression level of *ARF4* was not affected in fruit aged 39 dpa, prior to the appearance of the blotchy phenotype. Therefore, the reduced level of *ARF4* is more likely to be ripening-related and not a result of co-suppression. We cannot exclude that *ARF4* could potentially be regulated by *ARF2A*, however, it appears that variable expression of *ARF* genes in the patches, is not responsible for the uneven ripening pattern. These results corroborate our conclusion that the blotchy fruit phenotype is a genuine process that occurs when the entire fruit exhibits equally high levels of the *ARF2A* gene expression.

### *ARF2-OX* fruit display earlier ethylene emission and their phenotype is ethylene-dependent

**Fig 5:**
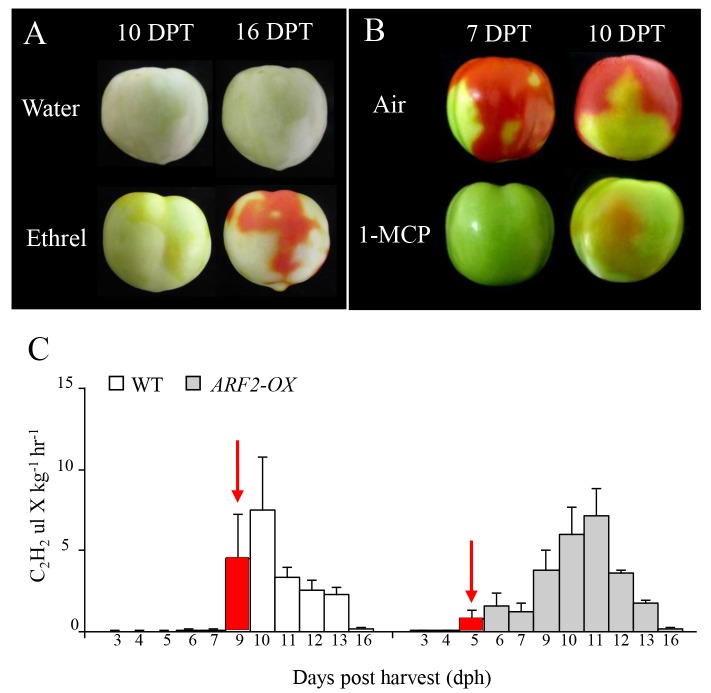
Altering ethylene signaling in *ARF2-OX* transgenic fruit. *ARF2-OX* fruit at the mature green (MG) stage, before the visual appearance of patches, were treated with either (A) ethrel, or (B) 1-MCP, and phenotypes were observed at (A) 10 and 16, or (B) 7 and 10 DPT. (C) Ethylene emission was measured from WT and *ARF2-OX* fruit harvested at the MG stage, every 1–3 days for 16 days, the red bars and arrows indicate the breaker stage. Error bars represent SE. DPT: days post treatment.

In order to examine the effects of ethylene on the *ARF2-OX* blotchy fruit phenotype, we treated fruit with either the ethylene releasing chemical ethrel or with the ethylene signaling inhibitor 1-MCP. Ethrel treatment enhanced the appearance of red, ripening regions (Fig 5A) while 1-MCP treatment delayed the appearance of red patches (Fig 5B). The overall pattern of the patches was not affected by the treatments but rather the rate of change in pigmentation. This implies that *ARF2-OX* fruit do exhibit normal ethylene-dependent ripening that can be accelerated or inhibited through ethylene manipulation, suggesting that the action of ARF2A is through the ethylene signaling pathway.

Ethylene emission was measured in WT and *ARF2-OX* fruit harvested at 39 dpa (MG stage) prior to the appearance of ripening regions. Interestingly, while the first clear rise in ethylene emission was observed at 9 days post-harvest (dph) in WT fruit, a rise in emission was seen four days earlier at 5 dph in the *ARF2-OX* fruit (Fig 5C). Emission of ethylene correlated with the initial observation of color change in fruit, further associating the accelerated blotchy ripening phenotype with ethylene emission and signaling.

### Transcriptome analysis of *ARF2-OX* fruit suggests acceleration of fruit ripening

**Fig 6:**
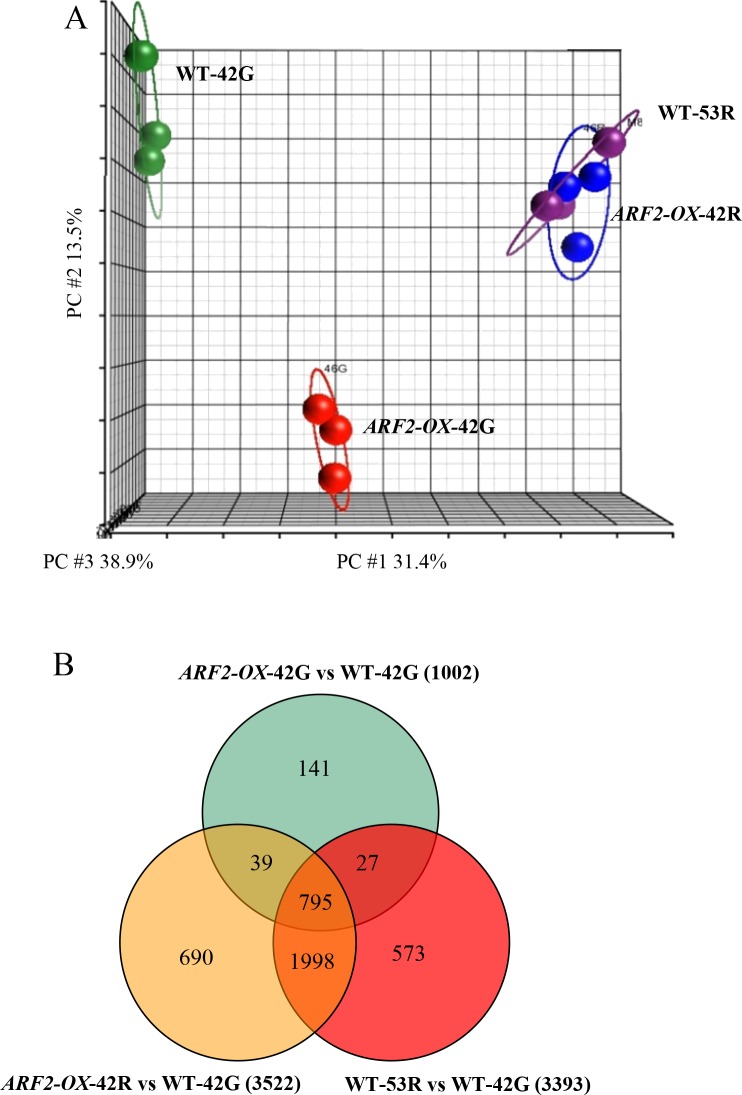
Microarray analysis of *ARF2-OX* fruit. Gene expression was analyzed in WT at 42 dpa (mature green stage; WT-42G) and 53 dpa (red stage; WT-53R) and green (*ARF2-OX*-42G) and red (*ARF2-OX*-42R) patches from *ARF2-OX* fruit at 42 dpa by microarray analysis. Results are displayed as (A) a principal component analysis (PCA) and (B) a Venn diagram of the differentially expressed genes in the comparisons: *ARF2-OX*-42G to WT-42G; *ARF2-OX*-42R to WT-42G; and WT-53R to WT-42G. P-value<0.01 and FDR<0.05; dpa: days post anthesis.

Transcriptome analysis was performed with samples from red and green regions of *ARF2-OX* 42 dpa fruit (*ARF2-OX-*42R and *ARF2-OX-*42G, respectively) as well as WT fruit at 42 dpa (WT-42G; MG stage) and 53 dpa (WT-53R; R stage; Fig 4E). Principal component analysis (PCA) indicated that *ARF2A* over-expression had a significant effect on the transcriptome of 42 dpa fruit not only in the red regions but also in the green ones (Fig 6A). Notably, the green tissues of WT (WT-42G) and *ARF2-OX-*42G fruit were separated from each other, indicating a difference already at this stage. The red region *ARF2-OX-*42R samples were projected together with the red WT ripening control samples (WT-53R) and both were projected apart from the WT-42G samples. Thus, the green patches in *ARF2-OX* lines are at a more advanced ‘ripening state’ than WT green fruit, but not as advanced as WT red fruit or *ARF2-OX* red patches.

We subsequently performed detailed analyses of differentially expressed genes comparing *ARF2-OX-*42G, *ARF2-OX-*42R and WT-53R to the WT-42G samples through a 5-way ANOVA analysis (S1 Table). Overall, 4263 genes were significantly up or down-regulated in one or more samples (Figs 6B and S7; S4 Table). A total of 1002, 3522 and 3393 genes were differentially expressed between *ARF2-OX-*42G, *ARF2-OX-*42R and WT-53R compared to the WT-42G samples, respectively. Using the set of 4263 differentially expressed genes we performed hierarchical clustering analysis (HCA; S7 Fig). Similarly to observations from the PCA using the global transcriptome data, the WT-53R and *ARF2-OX*-42R clustered together and away from the green samples in the HCA. Yet again, the *ARF2-OX*-42G sample exhibited an intermediary pattern between the WT-42G and the red region samples, as when the whole transcriptome data was used.

Of these 4263 differentially expressed genes, 2793 were differentially expressed in the *ARF2-OX* samples (either in the green, red or both regions) and in the red WT fruit, when compared to the WT-42G sample. The observation that the same genes exhibited changes in the expression levels also in the WT-53R sample, suggested that they could be ripening-related (Fig 6B). With the exception of six genes, changes in gene expression were in the same direction in all samples, *i*.*e*. genes were either all up-regulated (2098 genes) or all down-regulated (2159), not both. In addition, although the *ARF2-OX-*42R and WT-53R samples clustered together and shared more than two thirds of the differentially expressed genes, we found a large number of genes with differences in expression between them. This set might represent subtle differences in the ripening stage between these tissues, or non-ripening related genes that are associated with *ARF2A* over-expression.

We further characterized the set of 4263 differentially expressed genes by functional classification (S5 and S6 Tables). The distribution of Gene Ontology (GO) terms for each cluster was evaluated by functional enrichment analysis for carotenoid- and ethylene-related genes that could serve as markers of ripening (S8 and S9 Figs; S7 and S8 Tables). In the case of carotenoid-related genes, as expected most of the differentially expressed genes were up-regulated in red samples (*ARF2-OX-*42R and WT-53R) and not in *ARF2-OX-*42G (S8 Fig; S7 Table). An additional group of genes was up-regulated in the *ARF2-OX-*42G tissue which were mainly related to carotenoid cleavage and ABA biosynthesis. In the case of ethylene genes, HCA indicated that ethylene-related genes in the *ARF2-OX-*42G samples showed an intermediate expression level between the one in WT-42G fruit and the red samples (*ARF2-OX-*42R and WT-53R; S9 Fig; S8 Table).

**Fig 7:**
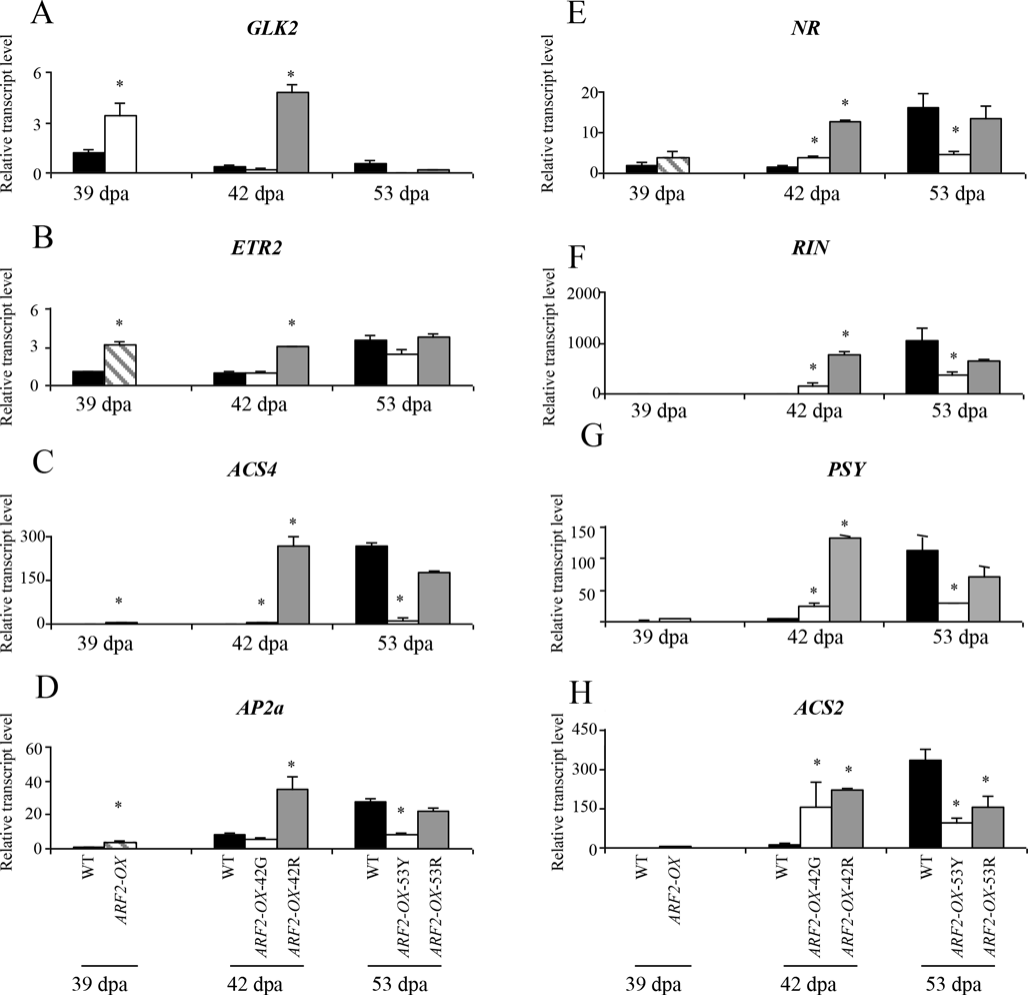
Expression of ripening-related genes in *ARF2-OX* fruit. Relative expression levels of ripening-related genes analyzed by qRT-PCR in WT and *ARF2-OX* fruit at 39, 42 and 53 dpa. (A) *GLK2*; (B) *ETR2*; (C) *ACS4*; (D) *AP2a*; (E) *NR*; (F) *PSY*; (G) *RIN*; (H) *ACS2*; Black bars represent WT, hatched bars *ARF2-OX* at 39 dpa, white bars *ARF2-OX* green patches at 42 and 53 dpa and grey bars *ARF2-OX* red patches at 42 and 53 dpa. Error bars represent SE. Statistical significance was evaluated using a student’s t-test, *p-value<0.05; dpa: days post anthesis.

The microarray results were validated for ripening-related genes and other genes of interest by qRT-PCR assays (either using the high-throughput Fluidigm qRT-PCR technology or standard assays). Several genes were significantly upregulated before the appearance of patches at 39 dpa in *ARF2-OX* green fruit, including *GOLDEN2-LIKE* (*GLK2*), *ETHYLENE RECEPTOR 2* (*ETR2*), *1-AMINOCYCLOPROPANE-1-CARBOXYLATE SYNTHASE 4 (ACS4*) and the ripening regulator *APETELA2a* (*AP2a*; Fig 7A–7D). At 42 dpa when the pigmented regions were visible and ripening advanced, *ACS4*, *ETR3* (*NEVER-RIPE*; *NR*), *PHYTOENE SYNTHASE* (*PSY*) and *RIPENING INHIBITOR* (*RIN*) were elevated in both regions (Fig 7C and 7E–7G), although to a greater extent in the red region, while *ACS2* was increased to a similar level in both green and red ones (Fig 7H). Table 1 summarizes the significant changes in expression of ripening-associated genes in *ARF2-OX-*42R (*i*.*e*. the red region of the transgenic fruit at 42 dpa) compared to the WT-42G samples (analyzed by microarray, Fluidigm or standard qRT-PCR assays). It can be seen that the majority of ripening-related regulators such as *RIN*, *AP2a*, *NOR*, *TAGL1*, *FRUITFULL-LIKE 1* (*FUL1*) and *GLK2* are upregulated as well as the ethylene biosynthesis genes *ACO1* (*1-AMINOCYCLOPROPANE-1-CARBOXYLATE OXIDASE 1*), *ACS2* and *ACS4*; ethylene receptors *ETR2*, *ETR3* (*NR*), *ETR4* and *ETR5*; and the carotenoid biosynthesis genes *PHYTOENE DESATURASE 1* (*PDS1*) and *PSY* (Table 1).

**Table 1:** Summary of expression profiles of ripening-related genes in red regions of the *ARF2*-*OX* fruit at 42 days post anthesis.

	Full Gene name	Short Name	Microarray	Fluidigm or qRT-PCR
**Ripening regulators**	*RIPENING INHIBITOR*	*RIN*	UP	UP
	*APETELA 2a*	*AP2a*	UP	UP
	*NON-RIPENING*	*NOR*	UP	▲
	*COLORLESS NON-RIPENING*	*CNR*	UP	⌘
	*TOMATO AGAMOUS-LIKE 1*	*TAGL1*	UP	UP
	*FRUITFULL 1*	*FUL1*	UP	▲
	*FRUITFULL 2*	*FUL2*	⌘	▲
	*GOLDEN-LIKE 2*	*GLK2*	⌘	UP
**Ethylene biosynthesis**	*1-AMINO-CYCLOPROPANE-1-CARBOXYLIC ACID OXIDASE 1*	*ACO1*	UP	UP
	*1-AMINO-CYCLOPROPANE-1-CARBOXYLIC ACID SYNTHASE 2*	*ACS2*	UP	UP
	*1-AMINO-CYCLOPROPANE-1-CARBOXYLIC ACID SYNTHASE 4*	*ACS4*	UP	UP
**Ethylene receptors**	*ETHYLENE RECEPTOR 1*	*ETR1*	⌘	▲
	*ETHYLENE RECEPTOR 2*	*ETR2*	⌘	UP
	*ETHYLENE RECEPTOR 3 (NEVER RIPE)*	*ETR3 (NR)*	UP	UP
	*ETHYLENE RECEPTOR 4*	*ETR4*	UP	⌘
	*ETHYLENE RECEPTOR 5*	*ETR5*	UP	▲
**Carotenoid biosynthesis**	*PHYTOENE SYNTHASE*	*PSY*	UP	UP
	*PHYTOENE DESATURASE 1*	*PDS1*	UP	UP

⌘ No significant change in gene expression

▲ Gene expression not analyzed

The transcriptome analysis complements and reinforces the previous findings in the *ARF2as* fruit that suggested a role for *ARF2A* in the regulation of tomato fruit ripening. They provide strong evidence that over-expression of *ARF2A* induces the ethylene-dependent ripening process at the transcriptional level, largely through the activation of ethylene signaling and master regulators associated with fruit ripening. It also suggests that the ripening-associated transcriptome is induced earlier in green regions than in WT green fruit, even prior to the visual appearance of distinct red and green regions.

### Fruit over-expressing *ARF2A* display altered ripening-associated specialized metabolism

**Fig 8:**
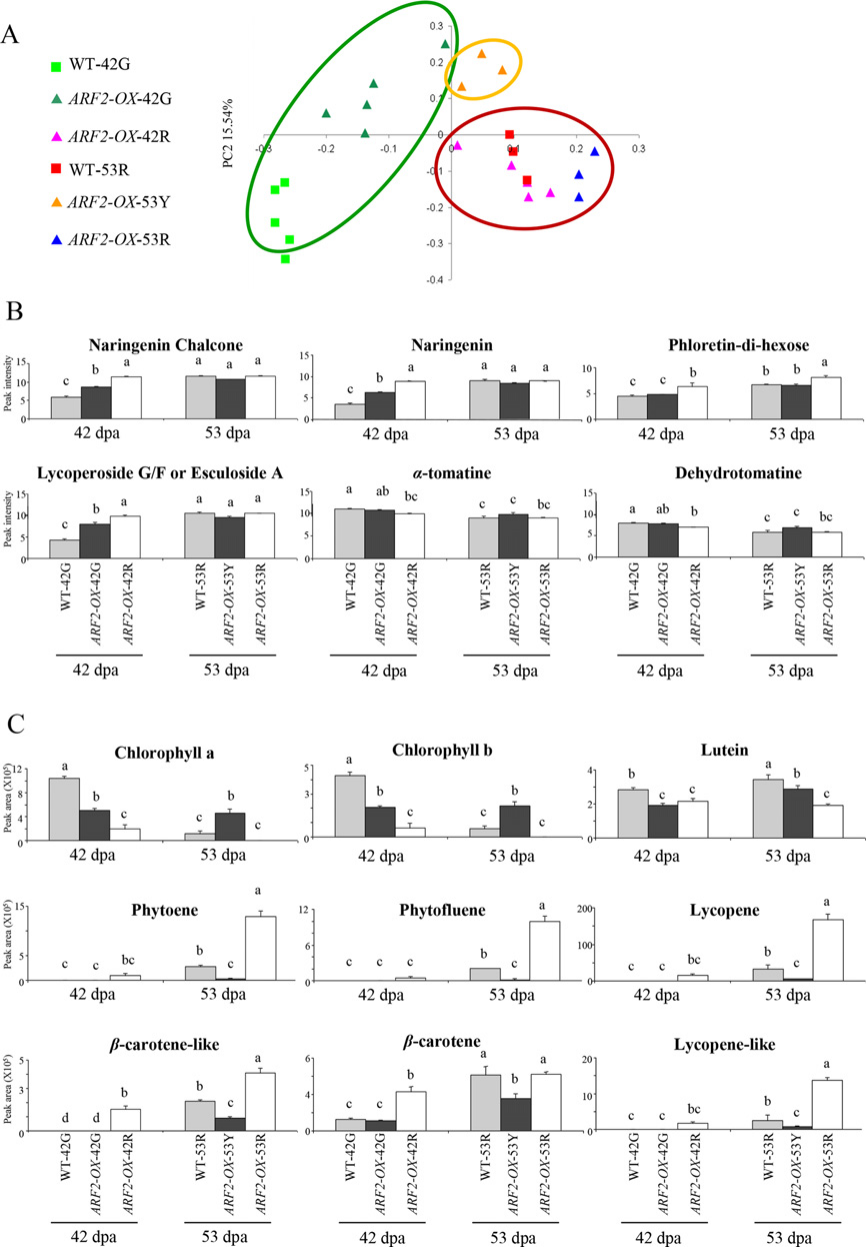
Metabolic analysis of *ARF2-OX* fruit. WT and *ARF2-OX* fruit were analyzed at 42 and 53 dpa by UPLC-qTOF-MS in positive mode, (A) results are visualized by a principle component analysis (PCA) plot; and displayed as histograms for (B) targeted flavonoids (upper row), and targeted glycoalkaloids (lower row). (C) Isoprenoids were analyzed in WT and *ARF2-OX* fruit at 42 and 53 dpa by HPLC. Grey bars represent WT, black bars *ARF2-OX* green/yellow patches and white bars *ARF2-OX* red patches. Error bars represent SE. Statistical significance was evaluated using a student’s t-test, *p-value<0.05; dpa: days post anthesis.

As changes in the production of secondary or specialized metabolites are a hallmark of the fruit ripening process, we carried out metabolic analysis of *ARF2-OX* fruit through a method that primarily detects semi-polar metabolites (mostly specialized metabolites) employing high-resolution mass spectrometry. A clear separation between the WT and transgenic fruit samples was observed in principle component analysis (PCA) of the metabolite data (Fig 8A; S9 and S10 Tables). The red pigmented samples (*i*.*e*. *ARF2-OX-*42R, *ARF2-OX*-53R and WT-53R) clustered together while the green ones (*i*.*e*. *ARF2-OX-*42G and WT-42G) were clearly separated, both from the red samples and from each other. The yellow sectors of the transgenic fruit (*ARF2-OX*-53Y) clustered separately, in between the red and green clusters.

Targeted analysis of selected flavonoids and glycoalkaloids was subsequently performed on the *ARF2-OX* fruit samples. Flavonoids generally accumulate during ripening as observed for naringenin chalcone, naringenin and phloretin-di-hexose in the WT-53R samples at as compared to WT-42G fruit (Fig 8B, upper row). In the *ARF2-OX* transgenic fruit, early accumulation of these flavonoids was observed in the red patches at 42 dpa. Some of the flavonoids exhibited early accumulation at 42 dpa in the green regions as well, but to a lesser extent than in the red ones. Most of the flavonoids examined in the transgenic fruit, either in the yellow or red patches, showed no difference at 53 dpa when compared to WT fruit. Similar results were obtained in the case of the glycoalkaloid lycoperoside (putatively lycoperoside G/F or esculeoside A) that typically accumulates in ripe fruit (Fig 8B, lower row; S11 Table). In contrast, the glycoalkaloids *α*-tomatine and dehydrotomatine, which are normally metabolized during fruit ripening, showed reduced levels in the red regions of the *ARF2-OX* fruit at 42 dpa. No significant differences in flavonoid levels were detected between the samples at 53 dpa fruit.

Analysis of isoprenoids, carotenoids and chlorophylls revealed overall similar results to those obtained with the flavonoids and glycoalkaloids (Fig 8C; S11 Table). Hence, metabolites typically accumulating in fruit ripening displayed early accumulation in the transgenic fruit, while those that decrease during WT ripening were reduced earlier than the WT fruit. Differences in the metabolite profiles in *ARF2-OX-*42G were minor as compared to those observed in *ARF2-OX-*42R (when compared to WT-42G fruit). In contrast to the flavonoid and glycoalkaloid profiles at 53 dpa, both regions of *ARF2-OX* fruit exhibited significant changes in isoprenoids, as compared to the WT red fruit (Fig 8C). The red regions of the transgenic fruit exhibited an intense ripening profile, while the yellow regions exhibited a delayed one, corresponding to the unripe WT fruit (WT-42G). The results demonstrated that overall, changes in specialized metabolism mirror those observed at the transcriptome level.

### Levels of hormones belonging to different classes are altered in *ARF2-OX* fruit

**Fig 9:**
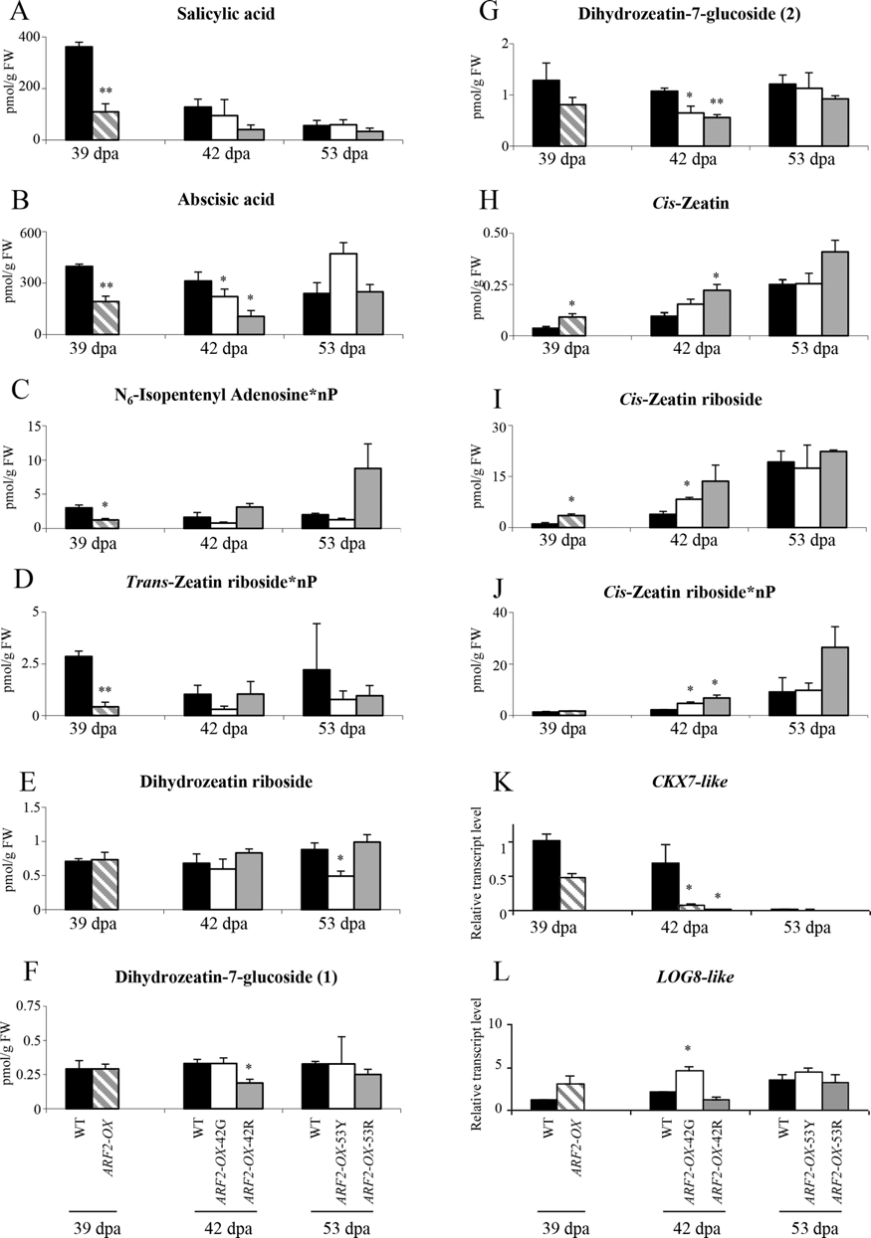
Hormone profiling in *ARF2-OX* transgenic fruit. WT and *ARF2-OX* fruit, at 39, 42 and 53 dpa were analyzed for levels of (A) salicylic acid; (B) abscisic acid; (C-G) cytokinins of the *trans*-zeatin biosynthesis branch and (H-J) cytokinins of the *cis*-zeatin biosynthesis branch, using UPLC-ESI-MS/MS. Relative expression level of the cytokinin-related genes (K) *CKX7-like*; (L) *LOG8-like* was analysed by qRT-PCR. Black bars represent WT, hatched bars *ARF2-OX* at 39 dpa, white bars *ARF2-OX* green patches at 42 and 53 dpa and grey bars *ARF2-OX* red patches at 42 and 53 dpa. Error bars represent SE. Statistical significance was evaluated using a student’s t-test, *p-value<0.05 and **p-value<0.01; dpa: days post anthesis.

The absolute concentrations of hormone metabolites belonging to different classes were measured in WT and *ARF2-OX* fruit (sampled at 39, 42 and 53 dpa, as previously described in Fig 4E). In total, 41 hormones were detected, 37 of which could be quantified, including: sixteen cytokinins (CKs), nine auxins (IAAs), five gibberellins (GAs), six abscisates (ABAs) and one salicylate (SA). Both abscisic acid and salicylic acid showed reduced accumulation in *ARF2-OX* fruit as early as at 39 dpa, prior to the appearance of the blotchy phenotype (Fig 9A and 9B; S12 Table). These hormones were also reduced during ripening in WT fruit. The reduced ABA level in *ARF2-OX* fruit is in correspondence with the high one observed in the *ARF2as* (Fig 3C). Remarkably, no significant differences were detected in the levels of auxins or gibberellins. However, a major hormone class that exhibited significant changes in *ARF2-OX* was the cytokinins. Cytokinins that represent the *trans*-zeatin biosynthesis branch including phosphorylated isopentenyl adenosine and phosphorylated *trans*-zeatin riboside were already reduced at 39 dpa (Fig 9C and 9D). Dihydrozeatin and dihydrozeatin-7-glucoside exhibited a reduction in the later stages (Fig 9E–9G). On the other hand, metabolites representing the *cis*-zeatin biosynthesis branch including *cis*-zeatin, *cis*-zeatin riboside, phosphorylated *cis*-zeatin riboside and *cis*-zeatin-7-glucoside, exhibited higher levels than the WT fruit (Fig 9H–9J). The *cis*-zeatin group of metabolites also accumulated upon ripening of the WT fruit. This may suggest a different role for the two isomers of zeatin during fruit ripening; and that the balance between *cis-* and *trans-*zeatin could be a target for ARF2A activity.

Since significant alterations in cytokinin levels were found in both *ARF2-OX* and in *ARF2as* lines (Figs 9C–9J and 3C), we mined the microarray data for changes in cytokinin-related genes (S4 Table). Two genes were significantly changed, *CYTOKININ OXIDASE* (*CKX*) responsible for cytokinin degradation (homolog of the Arabidopsis *CKX7*) and a *LONELY GUY* (*LOG*) involved in cytokinin production and activation (orthologue of Arabidopsis *LOG8*). *CKX7* was significantly downregulated in both patches of *ARF2-OX* fruit at 42 dpa, while *LOG8* showed an opposite expression profile being upregulated in 42 dpa in only the green patch (Fig 9I and 9J).

### Protein-protein interaction assays indicate that ARF2A homodimerizes and provides a further link to fruit ripening

**Fig 10:**
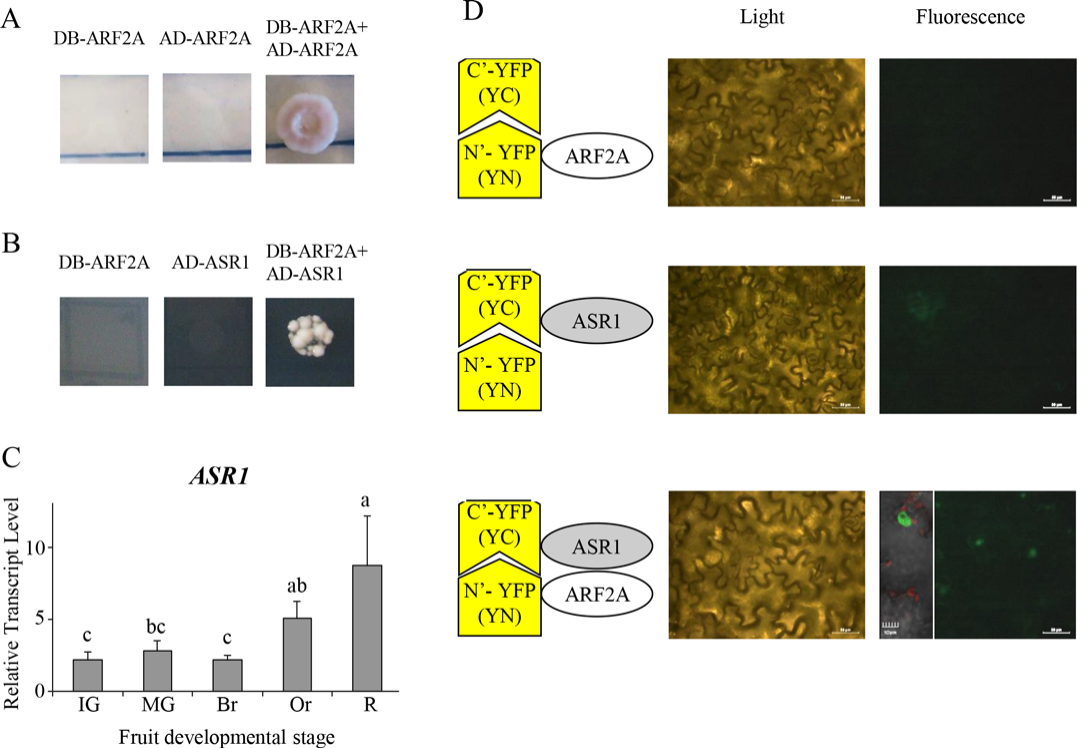
Dimerization of the ARF2A protein and its interaction. ARF2A was cloned downstream of the DNA-binding domain (DB-ARF2A) and co-transformed into yeast with either (A) ARF2A cloned downstream of the activation domain (AD-ARF2A); or (B) ASR1 cloned downstream of the activation domain (AD-ASR1), yeast growth on media lacking leucine, tryptophan, histidine and adenine indicated positive protein-protein interactions. (C) Relative expression levels of *ASR1* in WT cv. MicroTom fruit at five developmental stages (IG: immature green; MG: mature green; Br: breaker; Or: orange; and R: red), error bars represent SE; statistical significance was evaluated using an ANOVA test (JMP software, SAS) with three biological repeats based on the average of three technical replicates, values indicated by the same letter (a,b,c) are not statistically significant, p-value<0.05. (D) A Bimolecular Fluorescence Complementation assay (BiFC) was carried out by transient expression in tobacco leaves; ARF2A was cloned downstream of the amino-terminal region of YFP (yellow fluorescent protein; YN-ARF2A) and ASR1 was cloned downstream of the carboxy-terminal region of YFP (YC-ASR1); leaf regions were examined for fluorescent signal by light and confocal fluorescence microscopy. Inset zoom region shows that the ARF2A-ASR1 interaction is nuclear localized. Scale bars in the light and confocal fluorescence microscopy represent 50 μm and 10 μm, respectively.

ARF proteins comprise a DNA binding domain, a central region that can function as either an activation or repression domain and a dimerization domain [22]. The latter domain is known to interact with Aux/IAA proteins and other ARF proteins and it is accepted that ARFs function as either homo- or hetero-dimers with other ARFs [24]. We examined the possibility that ARF2A homo-dimerizes using a yeast-two-hybrid system. Growth of yeast on selection media confirmed that ARF2A indeed has the potential to homo-dimerize (Fig 10A) and that this may represent a true *in vivo* interaction.

To identify ARF2A interactors, we performed a yeast-two-hybrid screen using a library generated from fruit at several developmental stages (*i*.*e*. Breaker, Orange and Red). Among all interactions obtained only one exhibited an in-frame protein coding sequence that showed homology to the ABA STRESS RIPENING-INDUCED 1 (ASR1) protein (Fig 10B). This ASR family protein was shown previously to be ripening-related [43] and as we also observed in this study (Fig 10C). In order to confirm the interaction between ARF2A and ASR1, we conducted a bimolecular fluorescence complementation (BiFC) assay *in planta* [44, 45]. A positive interaction was observed between ARF2A and ASR1 as shown by the YFP fluorescence signal which was localized to the nucleus (Fig 10D). Taken together, these results suggest that ARF2A may homo-dimerize as well as interact with the ripening-associated ASR1 protein in the process of tomato fruit ripening.

## Discussion

### ARF2A is part of the regulatory network controlling tomato fruit ripening

Earlier work in tomato and Arabidopsis showed that ARF2A coordinates signaling cascades of light, ethylene and auxin in apical hook formation [31, 32]. Similar environmental and hormonal signaling pathways possibly play a significant role in the development and ripening of tomato fruit [8, 46]. The current study provides several lines of evidence showing that in tomato, ARF2A is likely a key element linking the ethylene signaling pathway to the ripening process. The expression pattern of *ARF2A* during the ripening process, in ripening-impaired mutants and upon blockage of ethylene signaling is one piece of this evidence. A different set of data supporting the primary indications was obtained through the detailed analysis of transgenic plants over-expressing *ARF2A* in tomato. A major outcome of *ARF2A* over-expression was a blotchy phenotype, which consisted of variegated green and red pigmented regions on the same fruit. The red regions exhibited an accelerated rate of ripening, as indicated by an early pattern of accumulation of ripening-related genes and metabolites. The upregulation of genes in *ARF2-OX* green fruit at 39 dpa, prior to the appearance of red pigmentation, suggested that ripening is accelerated even before the visual manifestation of the phenotype. Genes which are upregulated early in *ARF2-OX* lines include the major ethylene-related genes, *ETR2* and *ACS4*, and two transcription factors recently identified as ripening-regulators, *AP2a* [47] and *GLK2* (the *UNIFORM* gene) [48]. This is in line with a previous report on *ARF2* in Arabidopsis in which the expression of *ACS* genes was downregulated in the *arf2* mutant [27].

The influence of *ARF2A* over-expression on ripening was even more pronounced upon examination of transcriptomic changes occurring after the initiation of the ripening process, *i*.*e*. once red pigmentation appeared in particular regions (at 42 dpa). More than 2700 genes (out of the 4263 differentially expressed in the entire experiment) that exhibited a change of expression upon ripening in the WT fruit were also altered in both the green as well as the red regions of the transgenic fruit (prior to the initiation of pigmentation and ripening in the corresponding WT fruit). Indeed, we observed the induction of many genes considered to be ripening hallmarks in both green and red fruit regions including ripening regulators (e.g. *RIN*, *NOR*, *AP2a*, *TAGL1* and *FUL1*) as well as ethylene biosynthesis and signaling, particularly the receptors (*ACO1*, *ACS2*, *ACS4*, *ETR2*, *ETR3* (*NR*), *ETR4* and *ETR5* genes).

### Silencing of *ARF2* in tomato also delineates its involvement in the regulatory network controlling tomato fruit ripening

To complement the results obtained by analyzing the *ARF2-OX* fruit we generated *ARF2* silenced lines. Fruit of the *ARF2as* lines were parthenocarpic and exhibited delay in ripening. In line with our findings with *ARF2A* over-expression, knockdown of both *ARF2* homologs (*ARF2A* and *ARF2B*) resulted in changes to the profiles of ripening-associated transcription factors genes. Expression of *RIN*, *NOR*, *AP2a* and *TAGL1*, was induced in our study when *ARF2A* was over-expressed while transcripts of these master regulators were down-regulated in the *ARF2as*. In addition, alterations in the ripening process were also demonstrated by changes in the metabolic profile of the *ARF2as* red fruit. A parallel study [33] also investigated the consequences of down-regulating *ARF2A* and its homolog *ARF2B* in tomato fruit. Confirming our findings, the authors report that down-regulation of *ARF2* phenocopies features of previously identified non-ripening mutants including enhanced fruit firmness, low ethylene production and inability to ripen upon exogenous application of ethylene. The authors suggested that the reduced expression of the ripening regulators in knockdown lines were likely the main reason for the observed ripening defects. Taking these two complementary studies into consideration, the data suggests that ARF2A exerts its control of fruit ripening by controlling master regulators of the ripening in an ethylene signalling and biosynthesis-dependant manner.

### Blotchy fruit ripening and the ARF2A protein mode of action

Detailed molecular investigation of the *ARF2A* endogenous and transgene transcripts demonstrated that the blotchy ripening phenotype was due to a genuine over-expression of *ARF2A* in the transgenic fruit. Hence, clarifying the physiological and molecular mechanisms behind this phenotype might shed light on the ARF2A protein mode of action. In fact, during the regular development of tomato and additional fruit species, ripening does not ensue at once all over the fruit (as observed by the change of fruit pigmentation during the course of ripening). It is typically a gradual change which is probably driven by the auto-catalytic process of ethylene signaling in climacteric fruit. The phenotype we observed in *ARF2A* over-expressing fruit appears to represent acceleration as well as enhancement of what is seen in the regular process of fruit ripening in tomato. Blotchy ripening of fruit was described already some time ago in immature green fruit upon treatment with ethylene [49] and the authors suggested that the ability of ethylene to initiate ripening is dependent on the stage of the fruit. Here, we put forward the hypothesis that different areas on the same fruit are not synchronized in development and are more or less susceptible to ethylene and the induction to ripening. The hypothesis of differential sensitivity to ethylene signaling and ripening is strongly supported by the intermediate molecular and biochemical induction of the ripening in the green pigmented regions as compared to the WT green fruit and the red regions in the *ARF2A* over-expressing fruit. In other words, as we measured equal *ARF2A* transcripts in the green and red regions, fruit with tissues having similar sensitivity to ripen were not anticipated to show the blotchy phenotype observed here. Nevertheless, the putative molecular triggers that migrate across the fruit to form even ripening, and the blotchy phenotype that appears as a result of different conditions, should be addressed in future studies.

The intriguing question of what determines the level of the fruit tissue sensitivity to ethylene and ripening remains open. The knowledge of fruit ripening to date, as well results obtained in this study and the parallel publication [33] raise the hypothesis that the dose of active ethylene receptors could be a major factor that will determine the capacity of a particular fruit region to initiate ripening. With respect to this, it was reported that upon pathogen inoculation, ethylene was emitted from tomato leaves and the expression of ethylene receptors (*i*.*e*. *NR* and *ETR4*) were induced [50]. According to the authors, this reduces the sensitivity to ethylene in the tissue since ethylene signaling is a negative regulatory process and more ethylene receptors were synthesized. Thus, more ethylene would be required in order to inactivate the suppressors and to convey the ethylene signal. Moreover, in a different study, down‐regulation of the *ETR4* receptor produced an ethylene hypersensitive phenotype including accelerated ripening [51]. Here, the authors suggested that ETR4 monitors receptor levels and initiates the synthesis of new receptors as an ethylene response occurs, thus maintaining homeostasis in the ethylene response [51]. Furthermore, treatment of immature green fruit with ethylene, results in elevated levels of *NR* and *ETR4*, as well, affecting downstream ethylene signaling [52].

In this study, induction of the ethylene receptor *ETR2* was observed at 39 dpa in *ARF2-OX* fruit. This was followed by up-regulation of the *ETR3* (*NR*), *ETR4* and *ETR5* receptors at 42 dpa in the *ARF2-OX* fruit, prior to their induction in WT fruit. It is thus possible that this premature and uneven induction of the ethylene receptors (in the fruit tissue) contributes to the altered sensitivity to ethylene in different areas of the maturing fruit, resulting in the blotchy fruit phenotype.

### ARF2A is likely to play a role in the integration of hormonal cues during fruit ripening

The ARF2A protein was previously reported to be involved in many aspects of hormonal signaling pathways, in different plant species and organs [27, 29, 32, 53]. Both ABA and auxin were previously shown to stimulate ethylene emission in tomato fruit and were suggested to play a role in the regulation of fruit ripening [11, 15]. Here, we showed that in addition to being stimulated by ABA and auxin, *ARF2A* expression in fruit is induced by ethylene, albeit at a later time point than the ethylene biosynthesis gene *ACS4*. While all three hormone signaling pathways could be acting upstream of ARF2A, multi-class hormone and global transcriptome profiling suggests that ARF2A impacts ethylene biosynthesis and emission as well as ABA production. Both cytokinins and salicylic acid were also significantly altered in the *ARF2A* over-expression lines. In the *ARF2* silenced lines, a change in the levels of cytokinins and ABA, was observed. The two classes of cytokinins *i*.*e*. *cis*- and *trans*-zeatin metabolites are produced through separate metabolic pathways. Both were changed in an opposite manner, in the *ARF2-OX* fruit, however in the *ARF2as* fruit, both were reduced.

The ripening phenotype in *ARF2-OX* fruit could also be linked with cytokinin levels, as a blotchy ripening phenotype was previously observed in both fruit treated with exogenous cytokinin and lines over-expressing the *ISOPENTENYL TRANSFERASE* (*IPT)* gene which in turn, causes increased cytokinin levels [54]. Moreover, it has been previously proposed that the high levels of cytokinins observed in the tomato *rin* mutant contribute to its non-ripening phenotype and to inhibition of ripening-related processes [55]. Preliminary mining of the microarray data and expression assays of cytokinin biosynthesis genes showed that a *CYTOKININ OXIDASE 7* (*CKX7*) orthologue was significantly downregulated in both red and green regions at 42 dpa and a *LONELY GUY 8* (*LOG8*) ortholog (putatively encoding a cytokinin riboside 5’-monophosphate phosphoribohydrolase) was upregulated in the green region at 42 dpa. CKX enzymes have been shown to irreversibly catabolize cytokinins while LOG enzymes are involved in their biosynthesis [56]. Thus, simultaneously down regulating *CKX* and upregulating *LOG* would alter the levels of cytokinins as we indeed observed in the *ARF2A* over-expression fruit. The exact function of cytokinins in fruit ripening and with relation to ARF2A is far from being understood. Recently, [57] showed that ectopic *LOG1* expression in tomato creates a new hormonal balance and altered hormonal signaling that result in *de novo* formation of aerial minitubers from outgrowing juvenile tomato buds. Thus, it is not unconceivable that *LOG8* and *CKX7* could be involved in determining the specific hormonal balance required for the shift and induction of the fruit ripening process.

### ARF2A-ASR1 protein-protein interaction and the induction to ripening

A yeast-two-hybrid screen identified the ASR1 (ABA STRESS RIPENING-INDUCED 1) protein as a putative ARF2A interactor in tomato fruit. The interaction was further verified with a BiFC assay that localized the interaction to the nucleus. This finding is in agreement with previous studies that localized ASR1 to the nucleus where it acts as a transcription factor [58, 59]. In grape, an ASR protein was found to interact with an AP2-type transcription factor [60], strengthening the observation that ASR1 may act through interaction with other regulatory proteins, as here with ARF2A in tomato fruit. The ASR1 protein has been studied extensively and several studies proposed it to act as a component in the response to environmental signals, including ABA and glucose signaling, water deficiency as well as to integrate signaling pathways [43, 61–63]. Moreover, over-expression of the tomato *ASR1* in Arabidopsis resulted in an ABA-insensitive phenotype [64]. The ARF2A-ASR1 interaction consequently corresponds well with their similar expression profile during tomato fruit development and ripening, the ABA induced *ARF2A* expression in fruit and the dramatic decrease in ABA levels in the *ARF2-OX* fruit. The possibility that these two proteins could be coordinating the interaction between ethylene and ABA and the sensitivity of the fruit towards ethylene should be examined in future experiments.

**Fig 11:**
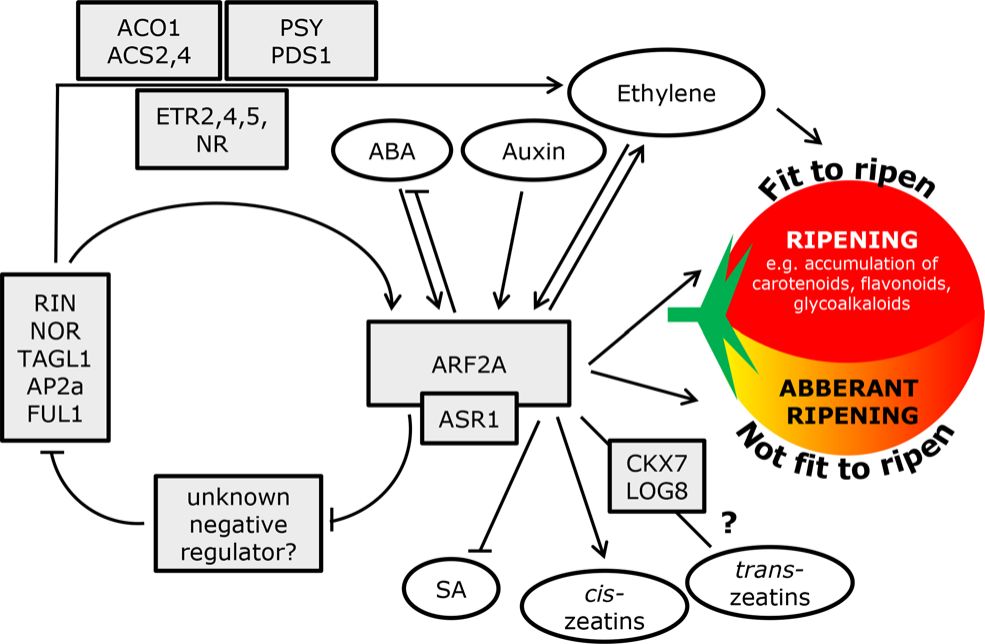
A general scheme depicting the activity of ARF2A in the regulatory network controlling fruit ripening. Data from this study and the complementary work [33] as well as previous reports concerning ripening control and information on tomato ARF2A and the ARF protein family was integrated and presented in a general scheme. ARF2A exerts its activity through the ethylene-dependent ripening pathway by impacting ripening regulators such as ones of the MADS-Box and AP2 protein families, genes associated with ethylene biosynthesis and signaling, carotenoid as well as other ripening metabolic pathways. The induction of ripening is likely indirect through an additional factor which functions as a ripening-repressor. ARF2A functions as a negative regulator, reducing the expression of the unknown ripening-repressor and thus activating the expression of several ‘master’ regulators and downstream ripening genes. It appears that ARF2A obtains signals from at least three hormone pathways, including ABA, auxin and ethylene while its activity impacts ABA, cytokinins and salicylic acid, at least at the level of hormone biosynthesis. The ASR1 protein likely interacts with ARF2A and together they fine tune the sensitivity of the fruit tissue to ethylene and to the capacity to ripen.

The amalgamation of data provided here and in the complementary study [33], as well as previous reports on tomato fruit ripening control and the ARF protein family, is presented in a form of a model scheme (Fig 11). ARF2A appears to play a key role in the induction of the ripening process in tomato. This factor exerts its activity through the widely studied ethylene-dependent ripening pathway by impacting genes currently considered as ‘markers’ of climacteric ripening including ethylene biosynthesis and signaling, carotenoid production and transcription factors of the MADS-box and AP2 protein families. *ARF2A* expression is significantly reduced in the *rin* and *nor* mutants and at the same time induced in the *TAGL1* over-expressing fruit, suggesting that it is part of a feedback mechanism existing in the ripening regulatory network. While this study implies that ARF2A has a positive impact on ripening, it was suggested to act as a negative regulator of transcription [20]. Thus, the activation of ripening could very well be indirect through an additional factor which functions as ripening repressor. It appears that ARF2A obtains input signals from at least three pathways, namely, ABA, auxin and ethylene. Furthermore, hormone profiling suggests that it impacts ABA, cytokinins and salicylic acid, at least in terms of biosynthesis.

Taken as whole, the ripening process in climacteric fruit is unquestionably dependent on ethylene; nevertheless, it requires significant input from several other hormone signaling pathways. The unique function of ARF2A in this composite interplay likely resides in its capacity to integrate signals and by doing so it enhances the sensitivity and thus readiness of the fruit tissue to ripen.

## Materials and Methods

### Plant material

Tomato plants (*Solanum lycopersicum*) cultivar (cv.) Ailsa Craig (AC) (obtained from the Tomato Genetics Resource Center; http://tgrc.ucdavis.edu), cv. MicroTom (obtained from Avi Levy, Department of Plant and Environmental Sciences, Weizmann Institute of Science, Israel) and cv. M82 (obtained from Yuval Eshed, Department of Plant and Environmental Sciences, Weizmann Institute of Science, Israel) were grown in a climate-controlled greenhouse at 24°C during the day and 18°C during the night, with natural light. The fruit stages used were immature green (IG), mature green (MG), breaker (Br), orange (Or) and red (R), which were harvested on average 10, 35, 38, 41 and 44 days post-anthesis in cv. MicroTom, respectively, unless otherwise mentioned. All fruit stages were harvested in three biological replicates (each replicate was represented by a pool of several fruit from different plants) and frozen in liquid nitrogen, placenta and seeds were removed.

### RNA extraction

Samples were ground into fine powder under liquid nitrogen and RNA was isolated as previously described [65] using 100 mg/ml extraction buffer (38% water-saturated phenol, 0.8M guanidine thiocyanate, 0.4M ammonium thiocyanate, 0.1M sodium acetate pH = 5, 5% glycerol). The aqueous phase was subsequently extracted twice with chloroform. Follow by isopropanol precipitation and two 70% ethenol washes, the RNA pellet was resuspended in DDW and stored at -20°C.

### Generation of transgenic tomato plants

The *ARF2-OX* expression cassette was generated by cloning of tomato *ARF2A* ORF into the pDRIVE vector (Qiagen, USA). Silent mutations were presented into the sequences by PCR. The mutated ORFs were cloned downstream of 35S CaMV promoter in pART7 vector and transferred to pART27 binary vector. For the *ARF2as* cassette, an approximately 1.5 kb fragment of the 5’ end of the *ARF2A* ORF was amplified. This fragment was cloned into NcoI/SacI sites in the pFLAP vector, in reverse complement orientation, downstream of 35S CaMV promoter. This fragment was transferred to the pBIN PLUS vector using PacI/AscI sites. Constructs were transformed to tomato plants, cv. M82, by *Agrobacterium tumefaciens* inoculation as previously described [66, 67].

### Ethylene and 1-MCP (1-Methylcyclopropene) and hormonal treatments

Fruit (*cv*. Ailsa Craig) harvested at the MG, Br and Or stages were incubated with 1 ppm of 1-MCP ("SmartFresh") for 19 h, moved to open air for 24 h, and subsequently frozen in liquid nitrogen. MG fruit (cv. MicroTom) were incubated in 40 ppm ethylene for 16 h, followed by 8 h of aeration at room temperature before snap freezing in liquid nitrogen. Control fruit were incubated in air instead of ethylene or 1-MCP. For other hormones treatmnets, fruit (cv. MicroTom) harvested at the stage mentioned were injected with 0.15 ml of the following hormones: 0.1 mM abscisic acid (ABA), 0.1 mM 1-naphthaleneacetic acid (NAA), 0.4% ethrel or DDW as control, following immersion in the solution for 10 minutes. In the case of ABA, this represents an approximately ten-fold increase in the measured physiological concentration of this hormone. Treated fruit were incubated at room temperature for the time indicated and then collected into liquid nitrogen and kept at -80°C until extracted.

### Ethylene emission measurements

Harvested fruit at the mature green stage were kept for 1 day in 250 ml flasks at room temperature, flasks were sealed for 4 hours, and ethylene was subsequently measured in the headspace by sampling using a syringe through a septum in the flask lid. The flasks were left open for 20 hours each day. Ethylene measurements were carried out every 1–3 days for the period of 16 days, as previously described [68] with some modifications. Ethylene was measured with a Varian 3300 gas chromatograph (CA, USA) equipped with a flame ionization detector and stainless steel column (HayeSep-T; 100/120 mesh) held at 70°C with helium as the carrier gas. Carbon dioxide was measured with gas chromatograph GOW-MAC 580 (Bethlehem, PA, USA) equipped with a thermal conductivity detector with a stainless steel column (AllTech Chromosorb 101, 80/100 mesh) held at 55°C and helium as the carrier gas. Ethylene was identified based according to the retention time of standard. Levels of ethylene were calculated according to standard curve (R^2^ = 0.999) and using the following formula: C2H4 = [ethylene (ppm) X free volume (ml)] / [fruit weight (g) X 4 (hours)].

### Metabolite analysis

Hormone extraction was carried out as previously described [69, 70] with some modifications; briefly, 200 mg of ground frozen plant tissue was extracted at -20°C with methanol/water/formic acid, containing stable isotope labeled internal standards (IS). Hormones were purified and fractionated by SPE and detected by UPLC-ESI-MS/MS operated in MRM mode. Quantification of hormones was done against external calibration curves, using analyte/IS peak ratios. A detailed protocol is included in S1 Method. In addition, the reproducibility of this method was tested successfully by repeating the analysis in two different harvest seasons, in ten developmental stages of tomato fruit development and ripening (S10 Fig). Extraction and analysis of other metabolites reported here was performed as previously described [41].

### Gene expression analysis

Three biological replicates of each RNA sample (*ARF2-OX*-42G; *ARF2-OX*-42R; WT-G; and WT-R) were labeled and hybridized to the 34K gene EUTOM3 Exon array (http://www.eu-sol.net/science/bioinformatics/data-and-databases/all-databases) and data were analyzed as previously described [48]. For Reverse-transcription and quantitative real-time PCR (qRT-PCR) DNaseI-treated RNA was reverse-transcribed using the High Capacity cDNA reverse transcription kit (Applied Biosystems) and cDNA was used for qRT-PCR analysis using three biological repeats per fruit stage, with three technical repeats for each sample. Gene-specific oligonucleotides were designed using Primer Express 2 software (Applied Biosystems). Expression of the *CAC* gene was used as an endogenous control, with the exception of S9 Fig, where *TIP41* was used [71]. Primers used in these assays are detailed in S13 Table. Analysis of gene expression by qRT-PCR in the Fluidigm platform was carried out in the Biological Services Unit, Weizmann Institute of Science, Israel, according to the manufacturer’s protocol (using EvaGreen DNA binding dye for gene expression with the 48.48 and 96.96 Dynamic Array IFCs PN 100–1208 A5-Advanced Development Protocol). Primers used in this study are detailed in S13 Table.

### Yeast-two-hybrid assays

The *ARF2A* ORF was cloned into PacI/AscI sites in pENTR/D-TOPO, and was introduced into pDEST32 vector using an LR clonase reaction (Invitrogen, USA; pDEST32-ARF2). These vectors were transformed into PJ69-4α yeast strain [72] by standard LiAc transformation (Yeast protocols handbook, Invitrogen, USA, July 2009) and used as the bait. The cDNA library used as the prey represented genes expressed in tomato fruit from breaker ripening stage and onwards. The library was cloned in pDEST22 vectors which together with the yeast strains were obtained from Prof. Richard Immink (Plant Research International B.V., Wageningen, The Netherlands). The interaction screenings were carried out using standard manufacturer’s protocol (ProQuest Two-Hybrid System with Gateway Technology, Invitrogen, USA), by two step transformation of the bait and prey vectors. Positive interactions were determined as positive growth on SD medium lacking leucine, tryptophan, histidine and adenine. Positive interactions were verified in a one-on-one yeast-two-hybrid. The interactor was identified through sequencing of the prey vector.

### Bimolecular fluorescence complementation (BiFC) assay

Analysis of protein-protein interaction was carried out by bimolecular fluorescence complementation (BiFC) assay as previously described [44, 45]. The *ARF2A* ORF was cloned into SalI/BamHI sites in the YN vector (YN-ARF2A), downstream of the sequence coding for the amino-terminus of the YFP fragment. The *ASR1* ORF was cloned into NdeI/BamHI sites in the YC vector (YC-ASR1), downstream of the sequence coding for the carboxy-terminus of the YFP fragment. The expression cassette from the YN and YC was extracted and cloned (using HindIII sites) into pCAMBIA vector and transformed to *A*. *tumefaciens*. The transient expression assay was carried out in one month old *N*. *tabacum* leaves as previously described [45]. Positive protein-protein interactions were identified as visualization of the YFP signal observed 48 h post-inoculation in fluorescent microscopy.

### Accession numbers

Sequence data from this article can be found in the EMBL/GenBank data libraries under the following accession numbers: *ARF2A* (Solyc03g118290; DQ340255), *ARF2B* (Solyc12g042070; HM143940), *ARF3* (Solyc02g077560; DQ340254), *ARF4* (Solyc11g069190; DQ340259), *ARF9* (Solyc01g096070; XM_010328837.1), *ASR1* (Solyc04g071610; AK326001.1), Arabidopsis *ARF2* (At5g62000; gi|30697610), *CNR* (Solyc02g077920; DQ672601), *RIN* (Solyc05g012020; AF448523), *TAGL1* (Solyc07g055920; AY098735), *FUL1* (Solyc06g069430; AY306155), *FUL2* (Solyc03g114830; AY306156), *AP2a* (Solyc03g044300; HQ586952), *NOR* (Solyc10g006880; AY573802), *GLK2* (Solyc10g008160; JX163897), *ACO1* (Solyc07g049530; EF501822), *ACS2* (Solyc01g095080; AY326958), *ACS4* (Solyc05g050010; NM_001247351), *ETR1* (Solyc12g011330; AF043084), *ETR2* (Solyc07g056580; AF043085), *ETR3* (*NR*; Solyc09g075440; AY600437), *ETR4* (Solyc06g053710; AY600438), *ETR5* (Solyc11g006180; AF118844), *ETR6* (Solyc09g089610; AY600440), *PSY* (Solyc03g031860; DQ335097), *PDS1* (Solyc03g123760; DQ339100), *CKX7* (Solyc08g061930; NM_001279287), *LOG8* (Solyc08g062820; XM_004245030), *ERF*.*E1/ERF2b* (Solyc09g075420; NM_001247379.2).

## Supporting Information

S1 Fig *ACS4* expression in altered ethylene treated fruit. Relative expression levels of *ACS4* in ethylene treated fruit at three developmental stages (MG, Br and R). Error bars represent SE. Statistical significance was evaluated using a student’s t-test, **p-value<0.01 and ***p-value<0.001; dpa: days post anthesis.
(TIF)

S2 Fig Alignment of *ARF2as* fragment with *ARF2A* and *ARF2B* sequences. Nucleotide alignment of the *ARF2as* construct with *ARF2A* and *ARF2B* genes, showing putative targeting of both closely related genes.
(PDF)

S3 Fig Expression levels of *ARF2* genes and fruit phenotypes in *ARF2as* transgenic lines. (A) Summary of *ARF2A* and *ARF2B* expression changes and fruit phenotypes in seven independent *ARF2as* transgenic lines. Relative expression levels of (B) *ARF2A* and (C) *ARF2B*, analyzed by qRT-PCR in red fruit of WT cv. MicroTom and independent *ARF2as* transgenic lines; error bars represent SE; statistical significance was evaluated using a student’s t-test with three biological repeats based on the average of three technical replicates, *p-value<0.05 and **p-value<0.01. Error bars represent SE; statistical significance was evaluated using a student’s t-test with three biological repeats, **p-value<0.01.
(TIF)

S4 Fig Gene expression analysis of *ARF2as* transgenic fruit. Relative expression levels of ripening regulators in *ARF2as* red fruit, analysed by qRT-PCR. DPA- days post anthesis; cv. MicroTom; * p-value<0.05; * *p-value<0.01
(TIF)

S5 Fig Expression levels of *ARF2A* and fruit phenotypes in different independent *ARF2-OX* transgenic lines. (A) Relative expression levels of *ARF2A* analyzed by qRT-PCR in leaves of WT cv. M82 and five independent *ARF2-OX* transgenic lines; error bars represent SE; statistical significance was evaluated using a student’s t-test with three biological repeats based on the average of three technical replicates, *p-value<0.05 and **p-value<0.01. (B) Summary of *ARF2A* expression changes and fruit phenotypes in eight independent *ARF2-OX* transgenic lines.
(TIF)

S6 Fig Expression analyses of *ARF2* homologs in *ARF2-OX* patches. Relative gene expression levels of ARF2 homologs (*ARF2B*, *ARF3*, *ARF4* and *ARF9*) in WT cv. M82 and *ARF2-OX* fruit at 39, 42 and 53 dpa. Error bars represent SE; statistical significance was evaluated using an ANOVA test (JMP software, SAS) with three biological repeats based on the average of three technical replicates, values indicated by the same letter (a,b,c) are not statistically significant, p-value<0.05; dpa: days post anthesis.
(TIF)

S7 Fig Hierarchical clustering analysis of *ARF2-OX* microarray data. Hierarchical clustering analysis of differentially expressed genes in *ARF2-OX* transgenic fruit, compared to WT-42G, as analyzed by microarray analysis.
(TIF)

S8 Fig Hierarchical clustering analysis of carotenoid-related genes in *ARF2-OX* fruit. Hierarchical clustering analysis of carotenoid-related genes in *ARF2-OX* transgenic fruit, compared to WT-42G, as analyzed by microarray analysis.
(TIF)

S9 Fig Hierarchical clustering analysis of ethylene-related genes in *ARF2-OX* fruit. Hierarchical clustering analysis of ethylene-related genes in *ARF2-OX* transgenic fruit, compared to WT-42G, as analyzed by microarray analysis.
(TIF)

S10 Fig Hormone analysis during fruit development in two seasons of harvest. Hormone levels were measured in ten fruit developmental stages (immature green to red ripe; 1 to 10) in two sequential growing seasons (2014 and 2015). Error bars represent SD.
(TIF)

S1 Table PCA of untargeted flavonoid analysis in negative mode from UPLC-QTOF-MS analysis of *ARF2as*. (XLSX)

S2 Table Significantly different putative metabolites identified to in red *ARF2as* tomato fruit by UPLC/qTOF-MS in positive and negative modes. (XLSX)

S3 Table Hormone analysis of *ARF2as* fruit. (XLSX)

S4 Table Differentially expressed genes in *ARF2-OX* transgenic fruit from microarray analysis. (XLSX)

S5 Table Classification of differentially expressed genes in *ARF2-OX* transgenic fruit according to molecular function (by Gene Orthology annotation). (XLSX)

S6 Table Classification of differentially expressed genes in *ARF2-OX* transgenic fruit according to biological processes (by Gene Orthology annotation). (XLSX)

S7 Table Carotenoid-related genes differentially expressed in *ARF2-OX* fruit. (XLSX)

S8 Table Ethylene- related genes differentially expressed in *ARF2-OX* fruit. (XLSX)

S9 Table PCA of untargeted flavonoid analysis in negative mode from UPLC-QTOF-MS analysis. (XLSX)

S10 Table PCA of untargeted flavonoid analysis in positive mode from UPLC-QTOF-MS analysis of *ARF2-OX*. (XLSX)

S11 Table Isoprenoids, carotenoids and glycoalkaloid in *ARF2-OX* fruit from HPLC analysis. (XLSX)

S12 Table Hormone analysis of *ARF2-OX* fruit. (XLSX)

S13 Table List of oligonucleotides used for cloning, qRT-PCR and Fluidigm© analyses in this study. (XLSX)

S1 Method Detailed protocol of the hormone analysis. (PDF)

## References

[1] Spencer MS. Ethylene Metabolism in Tomato Fruit: I. Relationship of Ethylene Evolution to Fruit Respiration and Ripening. Canadian Journal of Biochemistry and Physiology. 1956;34(6):1261–70. 13374589

[2] Trainotti L, Pavanello A, Casadoro G. Different ethylene receptors show an increased expression during the ripening of strawberries: does such an increment imply a role for ethylene in the ripening of these non-climacteric fruits? Journal of Experimental Botany. 2005;56(418):2037–46. 10.1093/jxb/eri202 15955790

[3] Klee H, Giovannoni J. Genetics and Control of Tomato Fruit Ripening and Quality Attributes. Annual Review of Genetics. 2011;45(1):41–59. 10.1146/annurev-genet-110410-132507 22060040

[4] Trainotti L, Tadiello A, Casadoro G. The involvement of auxin in the ripening of climacteric fruits comes of age: the hormone plays a role of its own and has an intense interplay with ethylene in ripening peaches. Journal of Experimental Botany. 2007;58(12):3299–308. 10.1093/jxb/erm178 17925301

[5] Ji K, Kai W, Zhao B, Sun Y, Yuan B, Dai S, et al SlNCED1 and SlCYP707A2: key genes involved in ABA metabolism during tomato fruit ripening. Journal of Experimental Botany. 2014 10.1093/jxb/eru288 PMC4157709 25039074

[6] Coombe BG, Hale CR. The Hormone Content of Ripening Grape Berries and the Effects of Growth Substance Treatments. Plant Physiology. 1973;51(4):629–34. 16658384 10.1104/pp.51.4.629 PMC366320

[7] Harpster MH, Brummell DA, Dunsmuir P. Expression Analysis of a Ripening-Specific, Auxin-Repressed Endo-1,4-β-Glucanase Gene in Strawberry. Plant Physiology. 1998;118(4):1307–16. 9847104 10.1104/pp.118.4.1307 PMC34746

[8] Srivastava A, Handa A. Hormonal regulation of tomato fruit development: A molecular perspective. Journal of Plant Growth Regulation. 2005;24(2):67–82. 10.1007/s00344-005-0015-0 .

[9] Pandolfini T. Seedless Fruit Production by Hormonal Regulation of Fruit Set. Nutrients. 2009;1(2):168–77. 10.3390/nu1020168 22253976 PMC3257607

[10] Sun L, Sun Y, Zhang M, Wang L, Ren J, Cui M, et al Suppression of 9-cis-Epoxycarotenoid Dioxygenase, Which Encodes a Key Enzyme in Abscisic Acid Biosynthesis, Alters Fruit Texture in Transgenic Tomato. Plant Physiology. 2012;158(1):283–98. 10.1104/pp.111.186866 22108525 PMC3252109

[11] Zhang M, Yuan B, Leng P. The role of ABA in triggering ethylene biosynthesis and ripening of tomato fruit. Journal of Experimental Botany. 2009;60(6):1579–88. 10.1093/jxb/erp026 19246595 PMC2671613

[12] Jones B, Frasse P, Olmos E, Zegzouti H, Li Z, Latche A, et al Down-regulation of DR12, an auxin-response-factor homolog, in the tomato results in a pleiotropic phenotype including dark green and blotchy ripening fruit. Plant Journal. 2002;32(4):603–13. 10.1046/j.1365-313X.2002.01450.x . 12445130

[13] Guillon F, Philippe S, Bouchet B, Devaux M, Frasse P, Jones B, et al Down-regulation of an Auxin Response Factor in the tomato induces modification of fine pectin structure and tissue architecture. Journal of Experimental Botany. 2008;59(2):273–88. 10.1093/jxb/erm323 . 18267945

[14] Vendrell M. Relationship between internal distribution of exogenous auxins and accelerated ripening of banana fruit. Australian Journal of Biological Sciences. 1970;23(6):1133–&. .

[15] Sozzi G, Trinchero G, Fraschina A. Ethylene and glycosidase promotion in GA(3)- and IAA-treated tomato fruit (Lycopersicon esculentum Mill.). Journal of Plant Growth Regulation. 2000;19(3):359–68. .

[16] Negi S, Sukumar P, Liu X, Cohen J, Muday G. Genetic dissection of the role of ethylene in regulating auxin-dependent lateral and adventitious root formation in tomato. Plant Journal. 2010;61(1):3–15. 10.1111/j.1365-313X.2009.04027.x . 19793078

[17] Meir S, Philosoph-Hadas S, Sundaresan S, Selvaraj KSV, Burd S, Ophir R, et al Microarray Analysis of the Abscission-Related Transcriptome in the Tomato Flower Abscission Zone in Response to Auxin Depletion. Plant Physiology. 2010;154(4):1929–56. 10.1104/pp.110.160697 20947671 PMC2996037

[18] Lin Z, Zhong S, Grierson D. Recent advances in ethylene research. Journal of Experimental Botany. 2009;60(12):3311–36. 10.1093/jxb/erp204 . 19567479

[19] Audran-Delalande C, Bassa C, Mila I, Regad F, Zouine M, Bouzayen M. Genome-Wide Identification, Functional Analysis and Expression Profiling of the Aux/IAA Gene Family in Tomato. Plant and Cell Physiology. 2012;53(4):659–72. 10.1093/pcp/pcs022 . 22368074

[20] Zouine M, Fu Y, Chateigner-Boutin A, Mila I, Frasse P, Wang H, et al Characterization of the Tomato ARF Gene Family Uncovers a Multi-Levels Post-Transcriptional Regulation Including Alternative Splicing. Plos One. 2014;9(1). 10.1371/journal.pone.0084203 . PMC3888382 24427281

[21] Tiwari S, Hagen G, Guilfoyle T. Aux/IAA proteins contain a potent transcriptional repression domain. Plant Cell. 2004;16(2):533–43. 10.1105/tpc.017384 . 14742873 PMC341922

[22] Guilfoyle T, Hagen G. Auxin response factors. Current Opinion in Plant Biology. 2007;10(5):453–60. 10.1016/j.pbi.2007.08.014 . 17900969

[23] Chapman E, Estelle M. Mechanism of Auxin-Regulated Gene Expression in Plants. Annual Review of Genetics. 2009;43:265–85. 10.1146/annurev-genet-102108-134148 . 19686081

[24] Rogg L, Bartel B. Auxin signaling: Derepression through regulated proteolysis. Developmental Cell. 2001;1(5):595–604. 10.1016/S1534-5807(01)00077-6 . 11709180

[25] Williams L, Carles C, Osmont K, Fletcher J. A database analysis method identifies an endogenous trans-acting short-interfering RNA that targets the Arabidopsis ARF2, ARF3, and ARF4 genes. Proceedings of the National Academy of Sciences of the United States of America. 2005;102(27):9703–8. 10.1073/pnas.0504029102 . 15980147 PMC1172271

[26] Fahlgren N, Montgomery T, Howell M, Allen E, Dvorak S, Alexander A, et al Regulation of AUXIN RESPONSE FACTOR3 by TAS3 ta-siRNA affects developmental timing and patterning in Arabidopsis. Current Biology. 2006;16(9):939–44. 10.1016/j.cub.2006.03.065 . 16682356

[27] Okushima Y, Mitina I, Quach H, Theologis A. AUXIN RESPONSE FACTOR 2 (ARF2): a pleiotropic developmental regulator. Plant Journal. 2005;43(1):29–46. 10.1111/j.1365-313X.2005.02426.x . 15960614

[28] Hughes R, Spielman M, Schruff M, Larson T, Graham I, Scott R. Yield assessment of integument-led seed growth following targeted repair of AUXIN RESPONSE FACTOR 2. Plant Biotechnology Journal. 2008;6(8):758–69. 10.1111/j.1467-7652.2008.00359.x . 18643948

[29] Strable J, Borsuk L, Nettleton D, Schnable P, Irish E. Microarray analysis of vegetative phase change in maize. Plant Journal. 2008;56(6):1045–57. 10.1111/j.1365-313X.2008.03661.x . 18764925

[30] Lim P, Lee I, Kim J, Kim H, Ryu J, Woo H, et al Auxin response factor 2 (ARF2) plays a major role in regulating auxin-mediated leaf longevity. Journal of Experimental Botany. 2010;61(5):1419–30. 10.1093/jxb/erq010 . 20164142 PMC2837260

[31] Li H, Johnson P, Stepanova A, Alonso J, Ecker J. Convergence of signaling of differential cell growth pathways in the control in Arabidopsis. Developmental Cell. 2004;7(2):193–204. 10.1016/j.devcel.2004.07.002 . 15296716

[32] Chaabouni S, Jones B, Delalande C, Wang H, Li Z, Mila I, et al Sl-IAA3, a tomato Aux/IAA at the crossroads of auxin and ethylene signalling involved in differential growth. Journal of Experimental Botany. 2009;60(4):1349–62. 10.1093/jxb/erp009 . 19213814 PMC2657550

[33] Hao Y, Hu G, Liu M, Mila I, Frasse P, Bouzayen M, et al. Auxin Response Factor SlARF2, a new component of the regulatory mechanism controlling fruit ripening in tomato. 2015. 10.1371/journal.pgen.1005649 PMC4696797 26716451

[34] Itkin M, Heinig U, Tzfadia O, Bhide A, Shinde B, Cardenas P, et al Biosynthesis of Antinutritional Alkaloids in Solanaceous Crops Is Mediated by Clustered Genes. Science. 2013;341(6142):175–9. 10.1126/science.1240230 . 23788733

[35] Ito Y, Kitagawa M, Ihashi N, Yabe K, Kimbara J, Yasuda J, et al DNA-binding specificity, transcriptional activation potential, and the rin mutation effect for the tomato fruit-ripening regulator RIN. Plant Journal. 2008;55(2):212–23. 10.1111/j.1365-313X.2008.03491.x . 18363783

[36] Fujisawa M, Nakano T, Shima Y, Ito Y. A Large-Scale Identification of Direct Targets of the Tomato MADS Box Transcription Factor RIPENING INHIBITOR Reveals the Regulation of Fruit Ripening. The Plant Cell. 2013;25(2):371–86. 10.1105/tpc.112.108118 23386264 PMC3608766

[37] Itkin M, Seybold H, Breitel D, Rogachev I, Meir S, Aharoni A. TOMATO AGAMOUS-LIKE 1 is a component of the fruit ripening regulatory network. Plant Journal. 2009;60(6):1081–95. 10.1111/j.1365-313X.2009.04064.x . 19891701

[38] Vrebalov J, Pan IL, Arroyo AJM, McQuinn R, Chung M, Poole M, et al Fleshy Fruit Expansion and Ripening Are Regulated by the Tomato SHATTERPROOF Gene TAGL1. The Plant Cell. 2009;21(10):3041–62. 10.1105/tpc.109.066936 19880793 PMC2782289

[39] Lincoln J, Campbell A, Oetiker J, Rottmann W, Oeller P, Shen N, et al Le-ACS4, a fruit ripening and wound-induced 1-AMINOCYCLOPROPANE-1-CARBOXYLATE SYNTHASE gene of tomato (Lycopersicon-esculentum)—expression in Escherichia-coli, structural characterization, expression characteristics, and phylogenetic analysis. Journal of Biological Chemistry. 1993;268(26):19422–30. . 8366090

[40] Katz Y, Galili G, Amir R. Regulatory role of cystathionine-gamma-synthase and de novo synthesis of methionine in ethylene production during tomato fruit ripening. Plant Molecular Biology. 2006;61(1–2):255–68. 10.1007/s11103-006-0009-8 . 16786305

[41] Mintz-Oron S, Mandel T, Rogachev I, Feldberg L, Lotan O, Yativ M, et al Gene expression and metabolism in tomato fruit surface tissues. Plant Physiology. 2008;147(2):823–51. 10.1104/pp.108.116004 . 18441227 PMC2409049

[42] Giovannoni J. Molecular biology of fruit maturation and ripening. Annual Review of Plant Physiology and Plant Molecular Biology. 2001;52:725–49. 10.1146/annurev.arplant.52.1.725 . 11337414

[43] Maskin L, Gudesblat G, Moreno J, Carrari F, Frankel N, Sambade A, et al Differential expression of the members of the Asr gene family in tomato (Lycopersicon esculentum). Plant Science. 2001;161(4):739–46. 10.1016/S0168-9452(01)00464-2 .

[44] Bracha-Drori K, Shichrur K, Katz A, Oliva M, Angelovici R, Yalovsky S, et al Detection of protein-protein interactions in plants using bimolecular fluorescence complementation. Plant Journal. 2004;40(3):419–27. 10.1111/j.1365-313X.2004.02206.x . 15469499

[45] Ohad N, Shichrur K, Yalovsky S. The analysis of protein-protein interactions in plants by bimolecular fluorescence complementation. Plant Physiology. 2007;145(4):1090–9. 10.1104/pp.107.107284 . 18056859 PMC2151733

[46] Giovannoni J. Genetic regulation of fruit development and ripening. Plant Cell. 2004;16:S170–S80. 10.1105/tpc.019158 . 15010516 PMC2643394

[47] Karlova R, Rosin F, Busscher-Lange J, Parapunova V, Do P, Fernie A, et al Transcriptome and Metabolite Profiling Show That APETALA2a Is a Major Regulator of Tomato Fruit Ripening. Plant Cell. 2011;23(3):923–41. 10.1105/tpc.110.081273 . 21398570 PMC3082273

[48] Powell A, Nguyen C, Hill T, Cheng K, Figueroa-Balderas R, Aktas H, et al Uniform ripening Encodes a Golden 2-like Transcription Factor Regulating Tomato Fruit Chloroplast Development. Science. 2012;336(6089):1711–5. 10.1126/science.1222218 . 22745430

[49] Bondad N, Pantasti E. Ethrel-induced ripening of immature and mature green tomato fruits. Economic Botany. 1972;26(3):238–44. 10.1007/BF02861036 .

[50] Ciardi J, Tieman D, Lund S, Jones J, Stall R, Klee H. Response to Xanthomonas campestris pv. vesicatoria in tomato involves regulation of ethylene receptor gene expression. Plant Physiology. 2000;123(1):81–92. 10.1104/pp.123.1.81 . 10806227 PMC58984

[51] Tieman D, Taylor M, Ciardi J, Klee H. The tomato ethylene receptors NR and LeETR4 are negative regulators of ethylene response and exhibit functional compensation within a multigene family. Proceedings of the National Academy of Sciences of the United States of America. 2000;97(10):5663–8. 10.1073/pnas.090550597 . 10792050 PMC25885

[52] Liu M, Pirrello J, Chervin C, Roustan J-P, Bouzayen M. Ethylene Control of Fruit Ripening: Revisiting the Complex Network of Transcriptional Regulation. Plant Physiology. 2015;169(4):2380–90. 10.1104/pp.15.01361 26511917 PMC4677914

[53] Vert G, Walcher C, Chory J, Nemhauser J. Integration of auxin and brassinosteroid pathways by Auxin Response Factor 2. Proceedings of the National Academy of Sciences of the United States of America. 2008;105(28):9829–34. 10.1073/pnas.0803996105 . 18599455 PMC2474533

[54] Martineau B, Houck C, Sheehy R, Hiatt W. Fruit-specific expression of the A-tumefaciens ISOPENTENYL TRANSFERASE gene in tomato—effects on fruit ripening and defense-related gene expression in leaves. Plant Journal. 1994;5(1):11–9. 10.1046/j.1365-313X.1994.5010011.x .

[55] Davey J, Vanstaden J. Endogenous cytokinins in fruits of ripening and non-ripening tomatoes. Plant Science Letters. 1978;11(3–4):359–64. 10.1016/0304-4211(78)90023-8 .

[56] Kurakawa T, Ueda N, Maekawa M, Kobayashi K, Kojima M, Nagato Y, et al Direct control of shoot meristem activity by a cytokinin-activating enzyme. Nature. 2007;445(7128):652–5. 10.1038/nature05504 . 17287810

[57] Eviatar-Ribak T, Shalit-Kaneh A, Chappell-Maor L, Amsellem Z, Eshed Y, Lifschitz E. A Cytokinin-Activating Enzyme Promotes Tuber Formation in Tomato. Current Biology. 2013;23(12):1057–64. 10.1016/j.cub.2013.04.061 . 23746638

[58] Maskin L, Maldonado S, Iusem N. Tomato leaf spatial expression of stress-induced Asr genes. Molecular Biology Reports. 2008;35(4):501–5. 10.1007/s11033-007-9114-2 . 17602312

[59] Ricardi M, Guaimas F, Gonzalez R, Burrieza H, Lopez-Fernandez M, Jares-Erijman E, et al Nuclear Import and Dimerization of Tomato ASR1, a Water Stress-Inducible Protein Exclusive to Plants. Plos One. 2012;7(8). 10.1371/journal.pone.0041008 . PMC3416805 22899993

[60] Saumonneau A, Agasse A, Bidoyen M, Lallemand M, Cantereau A, Medici A, et al Interaction of grape ASR proteins with a DREB transcription factor in the nucleus. Febs Letters. 2008;582(23–24):3281–7. 10.1016/j.febslet.2008.09.015 . 18804467

[61] Cakir B, Agasse A, Gaillard C, Saumonneau A, Delrot S, Atanassova R. A grape ASR protein involved in sugar and abscisic acid signaling. Plant Cell. 2003;15(9):2165–80. 10.1105/tpc.013854 . 12953118 PMC181338

[62] Frankel N, Carrari F, Hasson E, Iusem N. Evolutionary history of the Asr gene family. Gene. 2006;378:74–83. 10.1016/j.gene.2006.05.010 . 16822623

[63] Saumonneau A, Laloi M, Lallemand M, Rabot A, Atanassova R. Dissection of the transcriptional regulation of grape ASR and response to glucose and abscisic acid. Journal of Experimental Botany. 2012;63(3):1495–510. 10.1093/jxb/err391 . 22140241

[64] Shkolnik D, Bar-Zvi D. Tomato ASR1 abrogates the response to abscisic acid and glucose in Arabidopsis by competing with AB14 for DNA binding. Plant Biotechnology Journal. 2008;6(4):368–78. 10.1111/j.1467-7652.2008.00328.x . 18363631

[65] Chomczynski P, Sacchi N. Single-step method of RNA isolation by acid guanidinium thiocyanate-phenol-chloroform extraction. 1987;162(1):156–9. 10.1006/abio.1987.9999 2440339

[66] Meissner R, Jacobson Y, Melamed S, Levyatuv S, Shalev G, Ashri A, et al A new model system for tomato genetics. The Plant Journal. 1997;12(6):1465–72.

[67] Meissner R, Chague V, Zhu Q, Emmanuel E, Elkind Y, Levy AA. A high throughput system for transposon tagging and promoter trapping in tomato. The Plant Journal. 2000;22(3):265–74. 10849344 10.1046/j.1365-313x.2000.00735.x

[68] Fallik E, Polevaya Y, Tuvia-Alkalai S, Shalom Y, Zuckermann H. A 24-h anoxia treatment reduces decay development while maintaining tomato fruit quality. 2003;29(2):233–6.

[69] Kojima M, Kamada-Nobusada T, Komatsu H, Takei K, Kuroha T, Mizutani M, et al Highly Sensitive and High-Throughput Analysis of Plant Hormones Using MS-Probe Modification and Liquid Chromatography–Tandem Mass Spectrometry: An Application for Hormone Profiling in Oryza sativa. Plant and Cell Physiology. 2009;50(7):1201–14. 10.1093/pcp/pcp057 19369275 PMC2709547

[70] Shabala S, Cuin TA, Dobrev P, Vankova R. Quantification of Abscisic Acid, Cytokinin, and Auxin Content in Salt-Stressed Plant Tissues. Plant Salt Tolerance Methods in Molecular Biology. 913: Humana Press; 2012 p. 251–61. 22895765 10.1007/978-1-61779-986-0_17

[71] Exposito-Rodriguez M, Borges A, Borges-Perez A, Perez J. Selection of internal control genes for quantitative real-time RT-PCR studies during tomato development process. BMC Plant Biology. 2008;8(1):131 10.1186/1471-2229-8-131 19102748 PMC2629474

[72] James P, Halladay J, Craig EA. Genomic Libraries and a Host Strain Designed for Highly Efficient Two-Hybrid Selection in Yeast. Genetics. 1996;144(4):1425–36. 8978031 10.1093/genetics/144.4.1425 PMC1207695

