# Supplementary figures and images for: AUXIN RESPONSE FACTOR 2 Intersects Hormonal Signals in the Regulation of Tomato Fruit Ripening
Source: PLoS Genet. 2016 Mar 9;12(3):e1005903. doi: 10.1371/journal.pgen.1005903 (PMC4784954; doi:10.1371/journal.pgen.1005903)

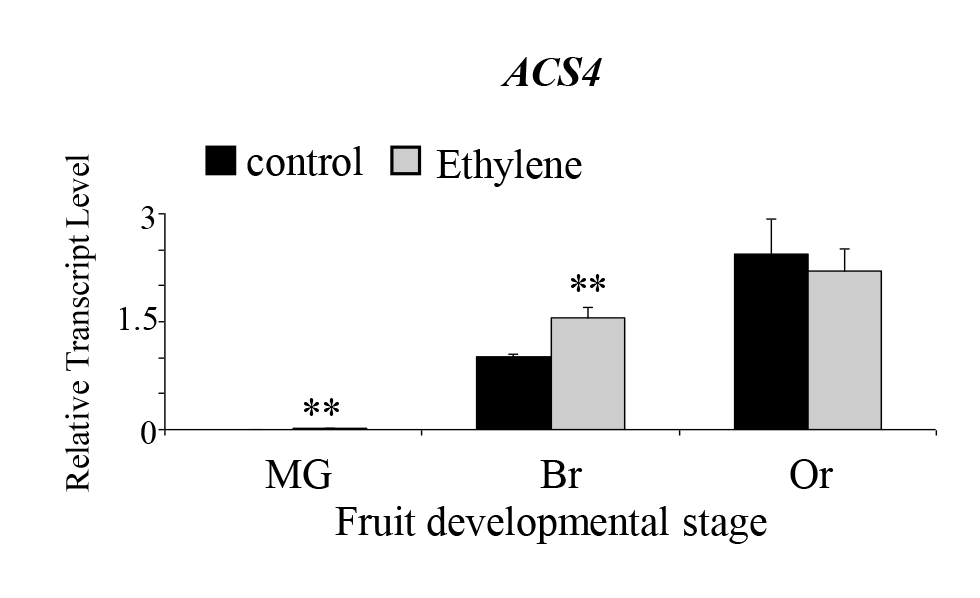

Supplement: S1 Fig — Relative expression levels of ACS4 in ethylene treated fruit at three developmental stages (MG, Br and R). Error bars represent SE. Statistical significance was evaluated using a student’s t-test, **p-value<0.01 and ***p-value<0.001; dpa: days post anthesis. (TIF) [file pgen.1005903.s001.tif]

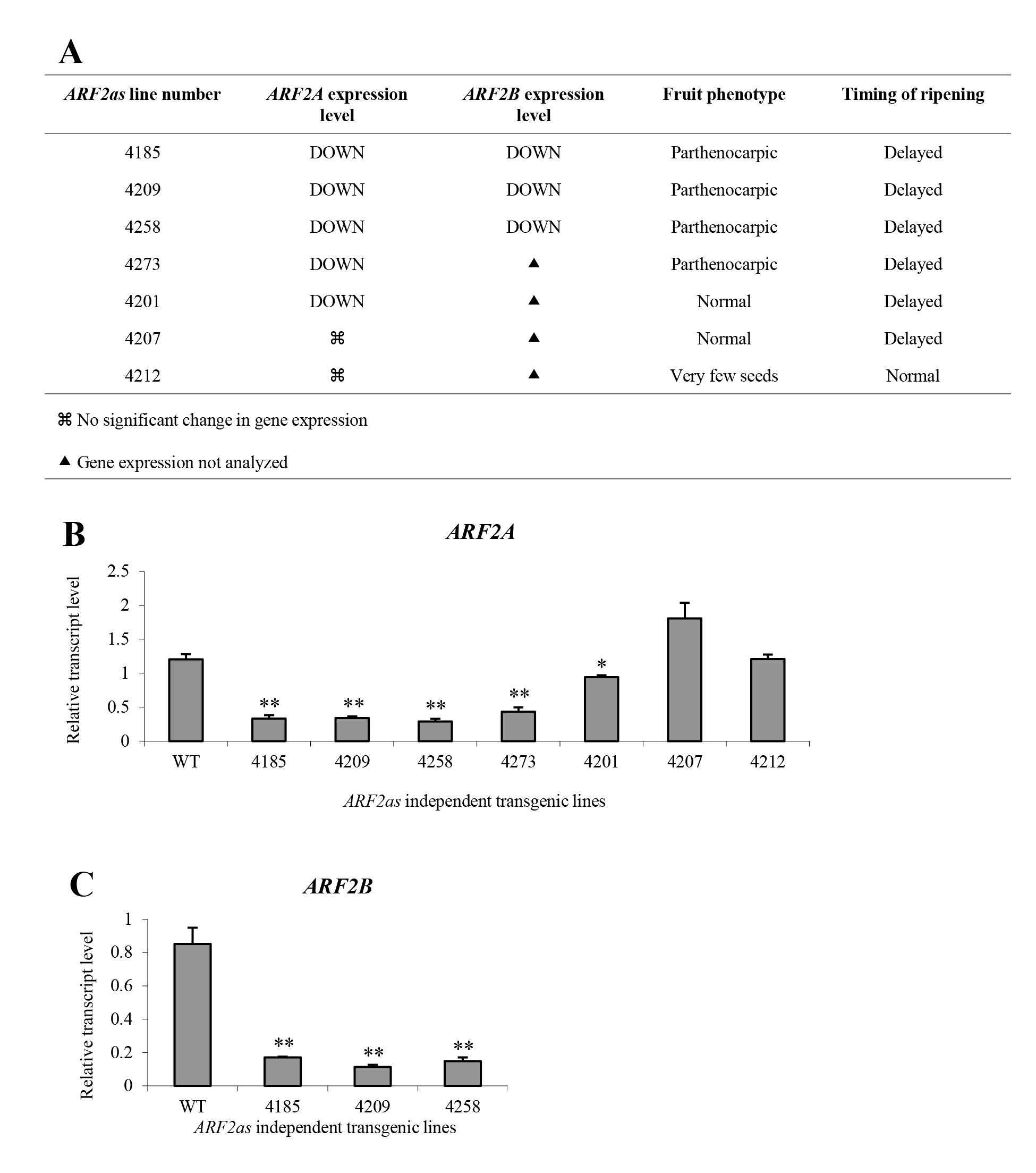

Supplement: S3 Fig — (A) Summary of ARF2A and ARF2B expression changes and fruit phenotypes in seven independent ARF2as transgenic lines. Relative expression levels of (B) ARF2A and (C) ARF2B, analyzed by qRT-PCR in red fruit of WT cv. MicroTom and independent ARF2as transgenic lines; error bars represent SE; statistical significance was evaluated using a student’s t-test with three biological repeats based on the average of three technical replicates, *p-value<0.05 and **p-value<0.01. Error bars represent SE; statistical significance was evaluated using a student’s t-test with three biological repeats, **p-value<0.01. (TIF) [file pgen.1005903.s003.tif]

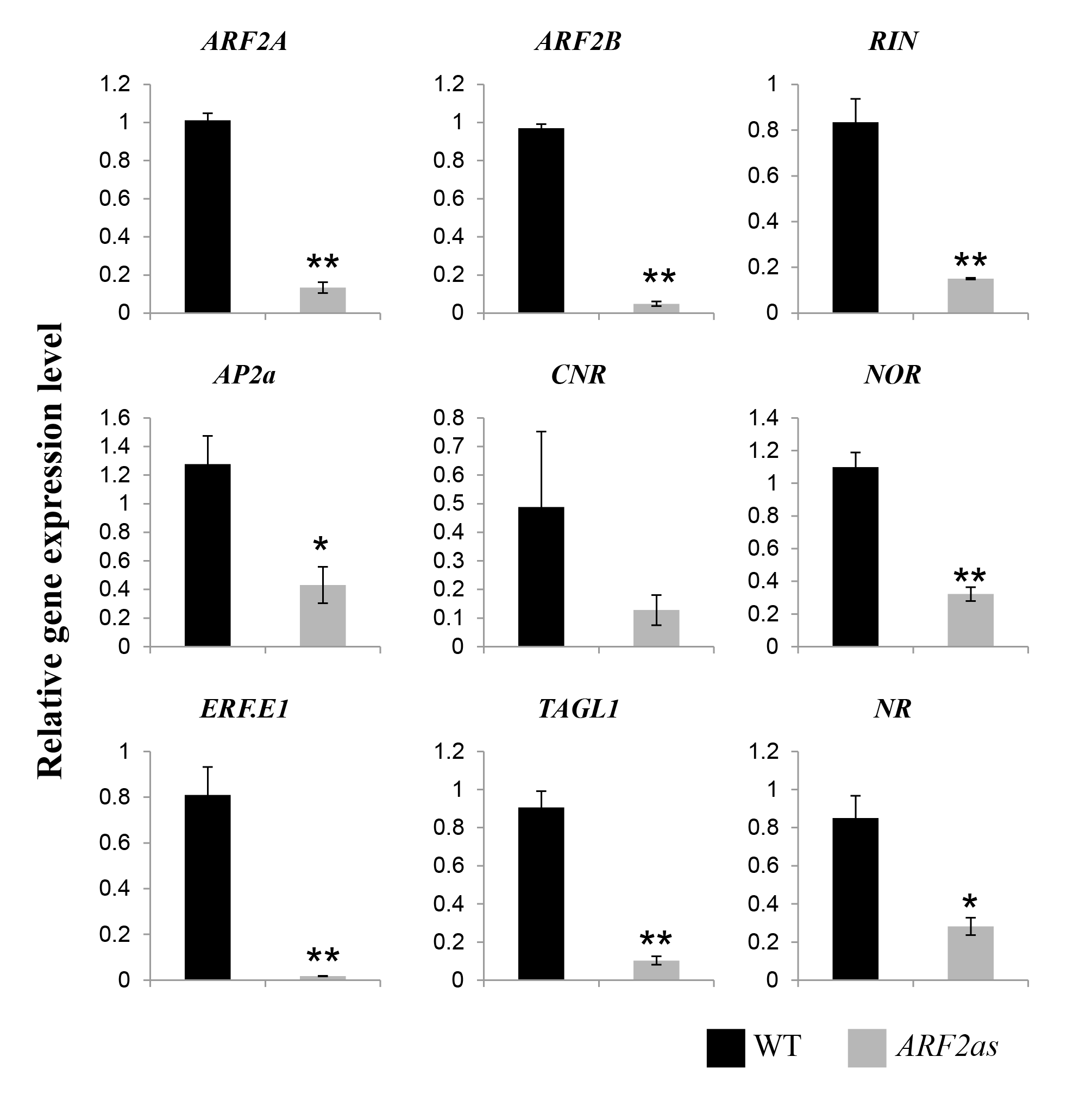

Supplement: S4 Fig — Relative expression levels of ripening regulators in ARF2as red fruit, analysed by qRT-PCR. DPA- days post anthesis; cv. MicroTom; * p-value<0.05; * *p-value<0.01 (TIF) [file pgen.1005903.s004.tif]

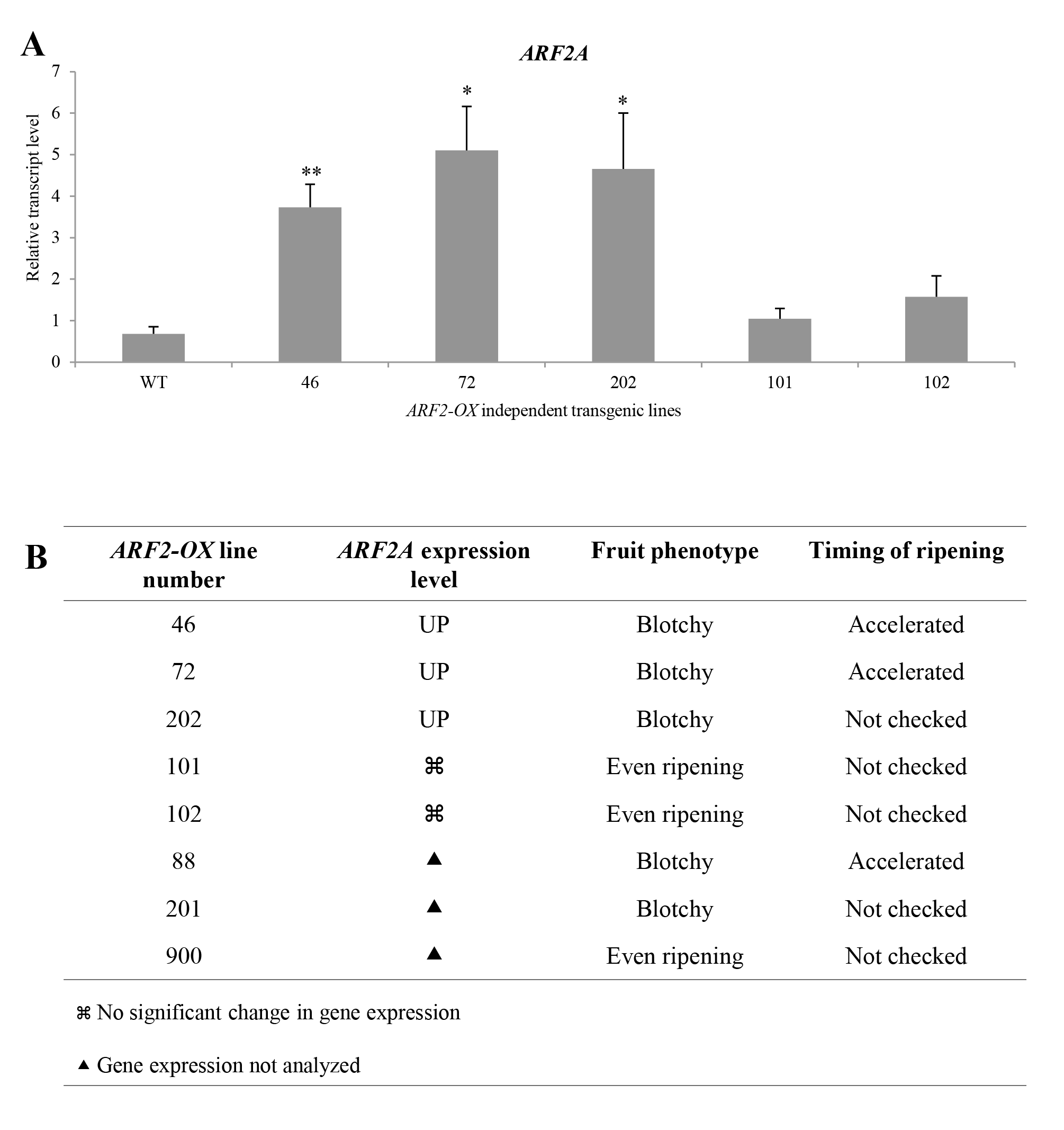

Supplement: S5 Fig — (A) Relative expression levels of ARF2A analyzed by qRT-PCR in leaves of WT cv. M82 and five independent ARF2-OX transgenic lines; error bars represent SE; statistical significance was evaluated using a student’s t-test with three biological repeats based on the average of three technical replicates, *p-value<0.05 and **p-value<0.01. (B) Summary of ARF2A expression changes and fruit phenotypes in eight independent ARF2-OX transgenic lines. (TIF) [file pgen.1005903.s005.tif]

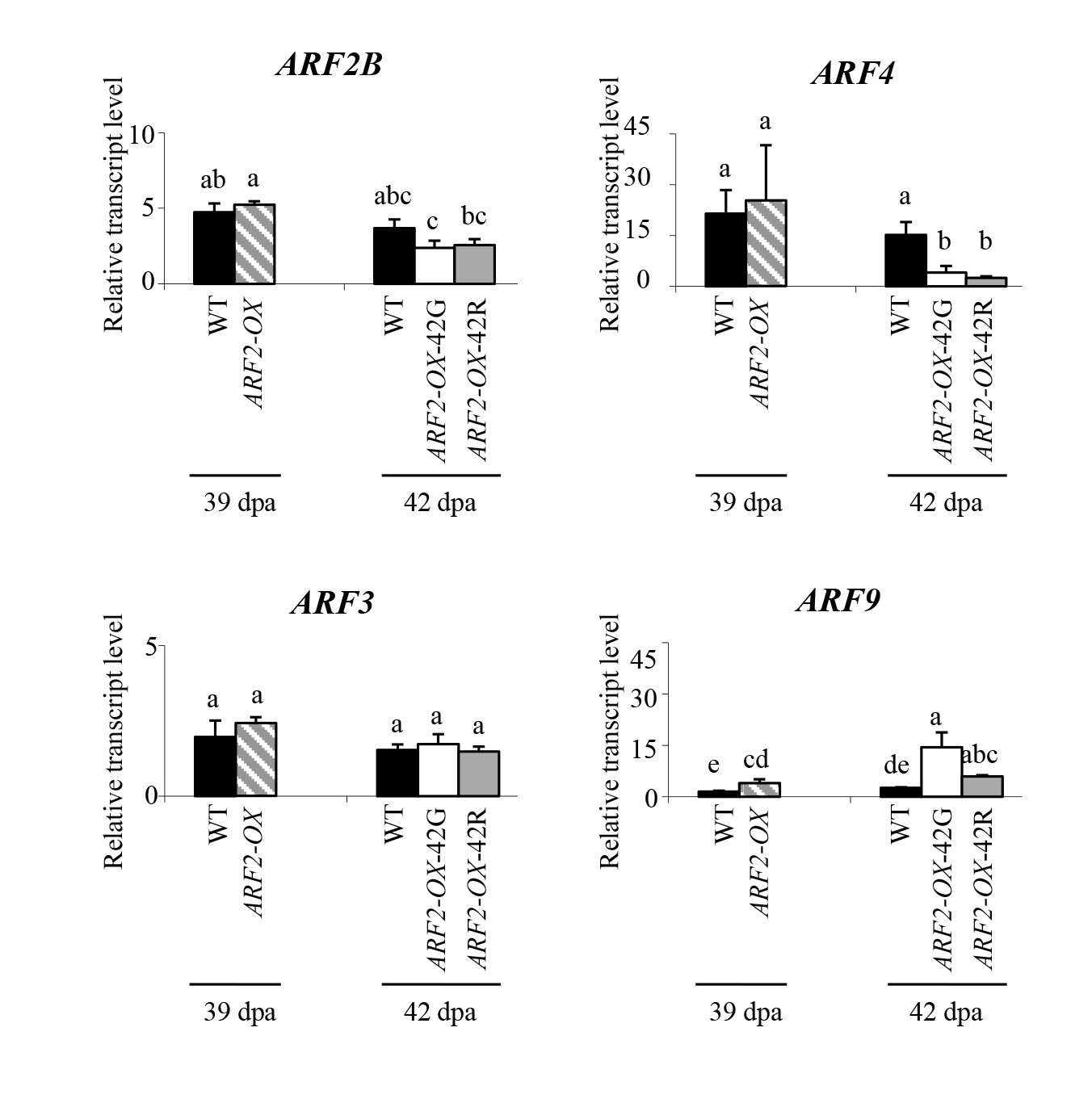

Supplement: S6 Fig — Relative gene expression levels of ARF2 homologs (ARF2B, ARF3, ARF4 and ARF9) in WT cv. M82 and ARF2-OX fruit at 39, 42 and 53 dpa. Error bars represent SE; statistical significance was evaluated using an ANOVA test (JMP software, SAS) with three biological repeats based on the average of three technical replicates, values indicated by the same letter (a,b,c) are not statistically significant, p-value<0.05; dpa: days post anthesis. (TIF) [file pgen.1005903.s006.tif]

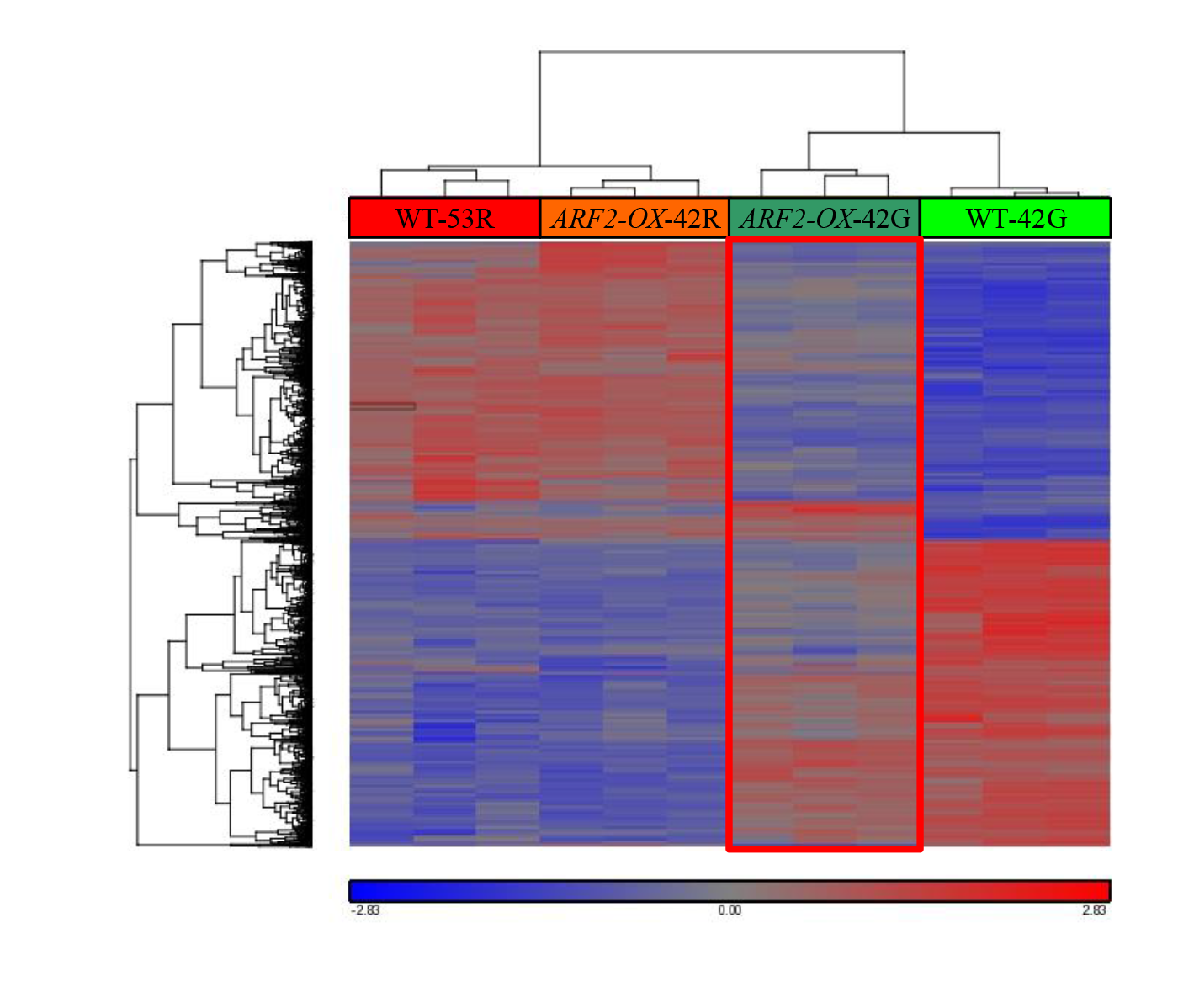

Supplement: S7 Fig — Hierarchical clustering analysis of differentially expressed genes in ARF2-OX transgenic fruit, compared to WT-42G, as analyzed by microarray analysis. (TIF) [file pgen.1005903.s007.tif]

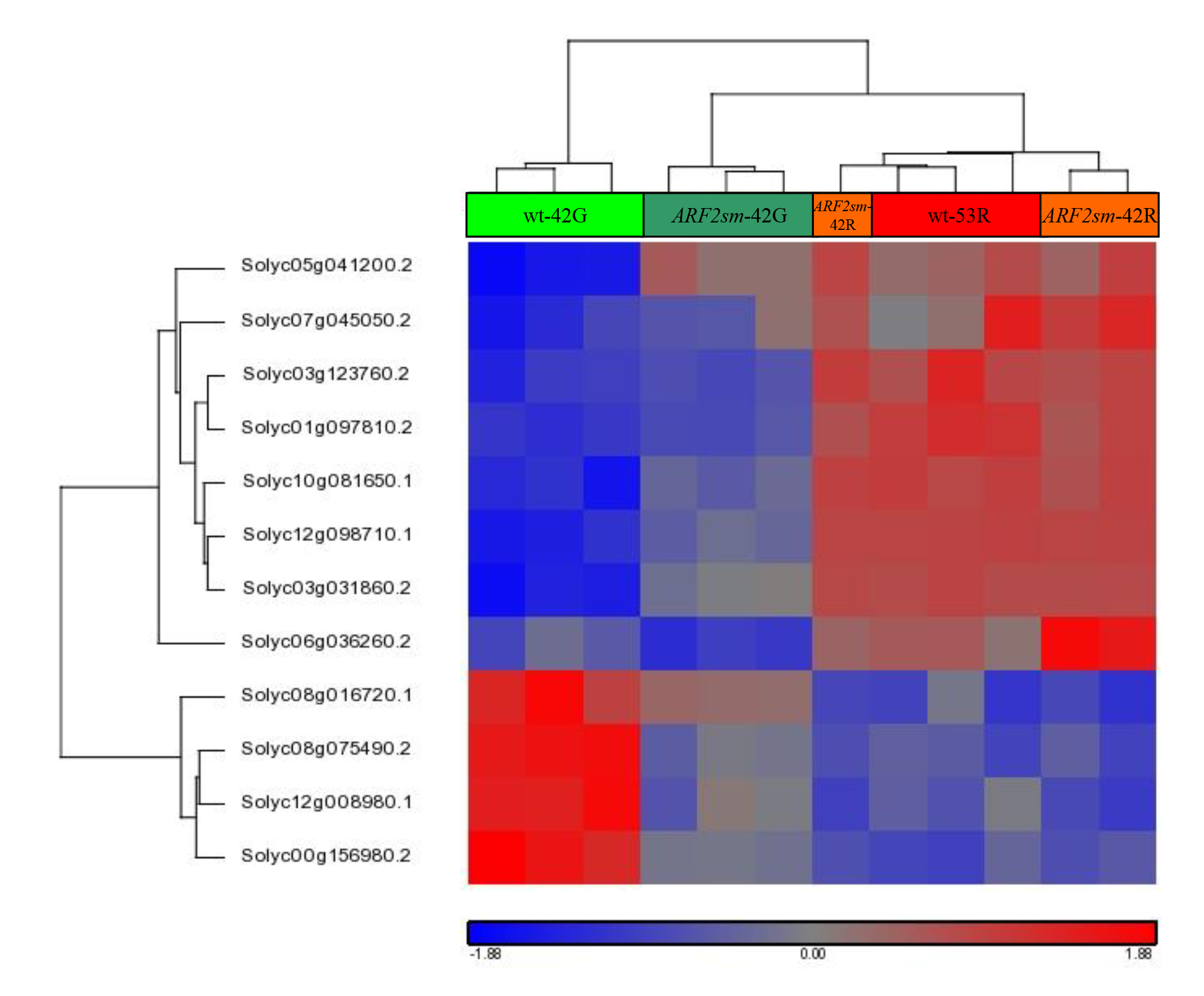

Supplement: S8 Fig — Hierarchical clustering analysis of carotenoid-related genes in ARF2-OX transgenic fruit, compared to WT-42G, as analyzed by microarray analysis. (TIF) [file pgen.1005903.s008.tif]

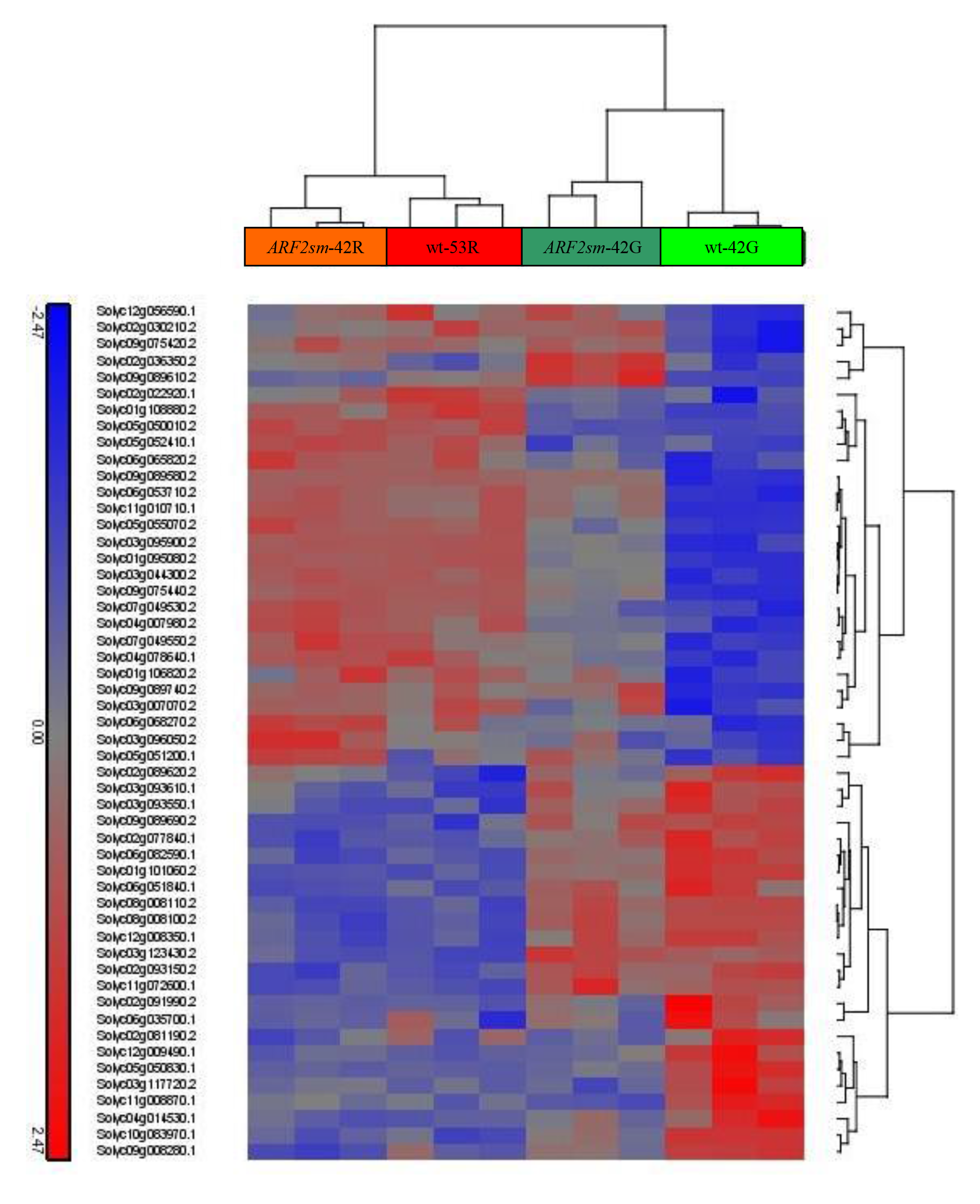

Supplement: S9 Fig — Hierarchical clustering analysis of ethylene-related genes in ARF2-OX transgenic fruit, compared to WT-42G, as analyzed by microarray analysis. (TIF) [file pgen.1005903.s009.tif]

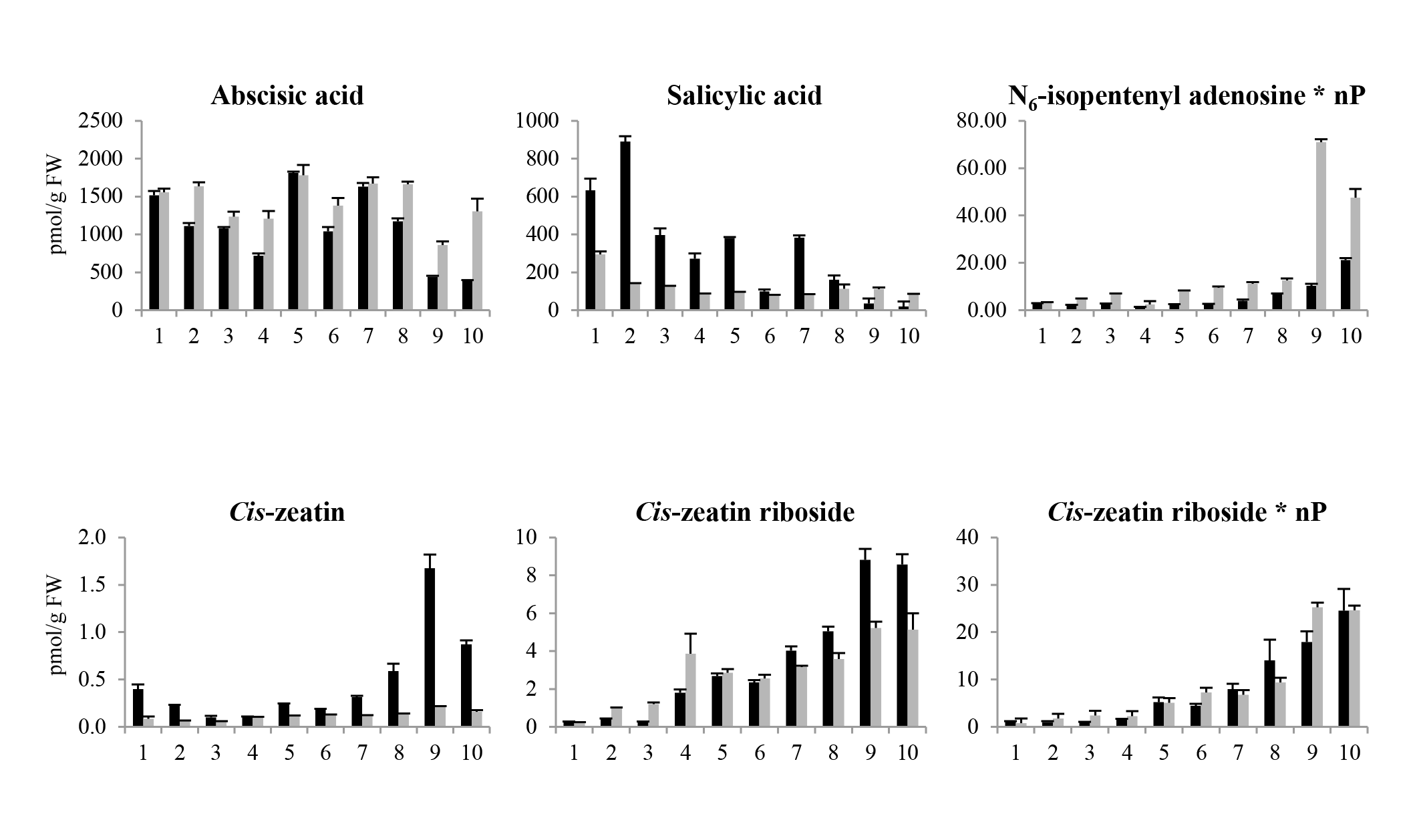

Supplement: S10 Fig — Hormone levels were measured in ten fruit developmental stages (immature green to red ripe; 1 to 10) in two sequential growing seasons (2014 and 2015). Error bars represent SD. (TIF) [file pgen.1005903.s010.tif]
