# Supplementary material for: AUXIN RESPONSE FACTOR 2 Intersects Hormonal Signals in the Regulation of Tomato Fruit Ripening
Source: PLoS Genet. 2016 Mar 9;12(3):e1005903. doi: 10.1371/journal.pgen.1005903 (PMC4784954; doi:10.1371/journal.pgen.1005903)
Supplement: S2 Fig — Nucleotide alignment of the ARF2as construct with ARF2A and ARF2B genes, showing putative targeting of both closely related genes. (PDF) [file pgen.1005903.s002.pdf]

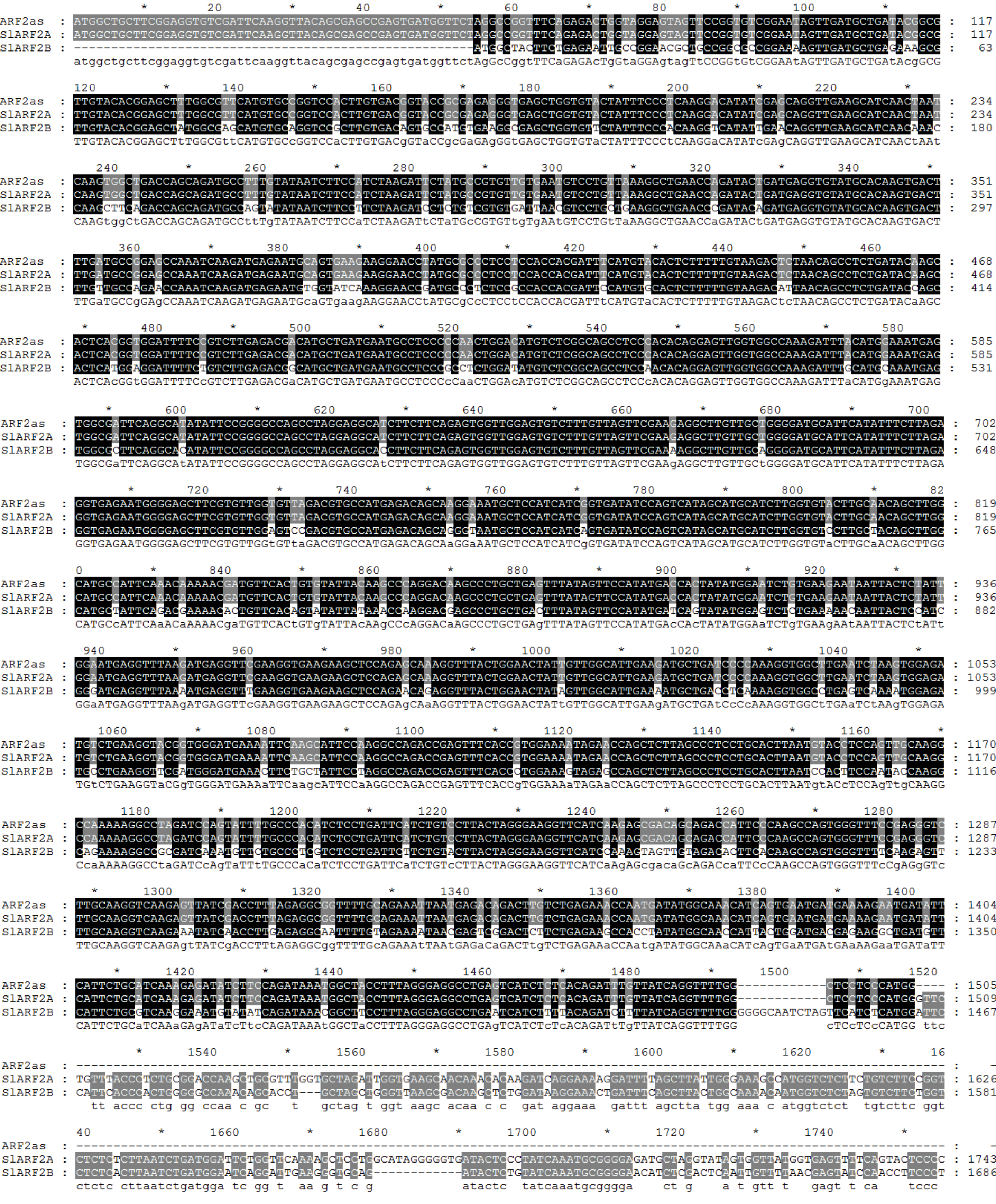
ARF2as
SlARF2A
SlARF2B

```
                1760         *        1780         *        1800         *        1820         *        1840         *        1860         *
ARF2as  : --------------------------------------------------------------------------------------------------------------------- :    -
SlARF2A : GGTCATAGGGTTGCAAATCAGCAGGGAAGCTGGATTATGCCTCAACCAGTGTCGCCTTACATGCAGCTCTCCTCTCATTCAAGAGAAATGATGCATAAACCCTCTGTAGTAAAGCAA : 1860
SlARF2B : GGTCATAGACCTGACAATCAGCAGGGAAATTGGTTAATGCCCCGTCCGTGCTGCCTTATATTCAGATGTCCGCTCATTCTGGAGAAATTATGCCTAAACCCATGGCTTCACCACAG : 1803
          ggtcatag   tg  aatcagcaggga  tgg t atgcc c   c gtg  gcctta at cag t tcc ctcattc  gagaaat atgc taaaccc   g    a   ca

                   1880         *        1900         *        1920         *        1940         *        1960         *        1980
ARF2as  : --------------------------------------------------------------------------------------------------------------------- :    -
SlARF2A : CCCGAGGCTGTGAAACCCAAAGAGGGTAACTACAAACTATTTGGCATTCCTCTTACAAGTAA---TGTTTGCACAGATGCTGTTATGATGCGGAAAAGTTCATTGATTGATCCAGCA : 1974
SlARF2B : CCCGAAGCCATGAAACCCAAAGAAGGGAACTGCAAACTATTTGGCATTCCCCTTGTAAGTAAATGTGCCACCATAGATCCTGTCATGTTGCGGAAAAATTCCCCGATTCACTCAACA : 1920
          cccga gc  tgaaacccaaaga gg aact caaactatttggcattcc ctt  aagtaa   tg    ca agat ctgt atg tgcggaaaa ttc   gatt a  ca ca

          *       2000         *        2020         *        2040         *        2060         *        2080         *        2100
ARF2as  : --------------------------------------------------------------------------------------------------------------------- :    -
SlARF2A : AGTGACATGAATATTGGTATACACCCTCATCAATCCCTTGCCACTGATTCTGATCAAAGGTCTGAGCAATCAAAGGGATCAAAGGTG---GATGATGGAGTTGCAGCTAATGATCAT : 2088
SlARF2B : AGTAACATGCACTTTGGTATACATCCACATCAATTCCCTATAATTGAATCTGATCAAAGGTCTGAGCAATCAAAGGGATCAAAGCTACCAGATGATGGCTTCATAGTTCATGATCAG : 2037
          agt acatg a  ttggtataca cc catcaat cc t   a tga tctgatcaaaggtctgagcaatcaaagggatcaaag t    gatgatgg  t   ag t atgatca

             *       2120         *        2140         *        2160         *        2180         *        2200         *        2220
ARF2as  : --------------------------------------------------------------------------------------------------------------------- :    -
SlARF2A : GACAAACAATTTCATACCTTTCATCTTGCTGCTAGAGATAAGGATGGCAAAGGGCATAGTAGTTCTACAAGGAGCTGCACAAAGGTTCATAAACAGGGTACAGCACTCGGAAGGTCC : 2205
SlARF2B : GAAGAACAATTCCAAACCTCTCATCCTGGTACTCGAGATAGAGAGGGCAAAGGCCTTGTTCATTCAACAAGGAGTTGCACCAAGGTTCATAAACAGGGTACAGCCCTTGGAAGGTCT : 2154
          ga  aacaatt ca acct tcatc tg t ct gagata  ga ggcaaagg c t  t  ttc acaaggag tgcac aaggttcataaacagggtacagc ct ggaaggtc

                *       2240         *        2260         *        2280         *        2300         *        2320         *        2340
ARF2as  : --------------------------------------------------------------------------------------------------------------------- :    -
SlARF2A : GTAGATCTTGCAAAGTTCAACAATTATGACGAATTGATTGCTGAACTGGATCAACTTTTTGATTTAATGGTGAACTCAAGGCTCGGAGTAAGAGTTGGCTGGTTGTATACACTGAT : 2322
SlARF2B : GTTGATCTTGCAAAGTTCAACAACTATGAAGAATTGATAGCTGAACTGGATCACATTTTTGATTTAATGGTGAGCTCAAGGCTCGTAACAAGAACTGGCTGGTTGTATATACTGAT : 2271
          gt gatcttgcaaagttcaacaa tatga gaattgat gctgaactggatca  tttttgatttaatggtga ctcaaggctcg a  aaga  tggctggttgtata actgat

                   *       2360         *        2380         *        2400         *        2420         *        2440         *       2
ARF2as  : --------------------------------------------------------------------------------------------------------------------- :    -
SlARF2A : GATGAGGGTGACATGATGCTTGTTGGAGATGATCCATGGCAGGAATTTTGTGGTATGGTTCGCAAGATTTTCATCTATACAAAAGAGGAGGTGCAGCGTATGAATCCAGGGACCCTT : 2439
SlARF2B : GATGAGGGTGACATGATGCTTGTTGGAGATGATCCATGG---GAATTTTGTGGTATGGTTCGGAAGATTTTTATCTACACGAAAGATGAGGTGCAGCGGATGAACCCTGGGACTCTC : 2385
          gatgagggtgacatgatgcttgttggagatgatccatgg   gaattttgtggtatggttcg aagatttt atcta ac aaaga gaggtgcagcg atgaa cc gggac ct

          460         *        2480         *        2500         *        2520         *        2540         *
ARF2as  : ------------------------------------------------------------------------------------------------------ :    -
SlARF2A : AATTCAAAAGGTGAGGACACTTCTTCTGTTGCTGAGGGCTCAGACGCTAAAGAAGTGAAGAATCTGCAACTTCCTTCTGAATCTGGTCAAGCAGAATCTTAG : 2541
SlARF2B : AATTCAAAAGGCGAGGACAATTCTTCTGTTGCAGAAGGCTCTGATGCTAAAGAAGTGAAGAATCTACAGCTTCACATTGATTCCAGTCCGGAAGATTCTTAG : 2487
          aattcaaaagg gaggaca ttcttctgttgc ga ggctc ga gctaaagaagtgaagaatct ca cttc    tga tc  gtc  g aga tcttag
```
