# Supplementary material for: AUXIN RESPONSE FACTOR 2 Intersects Hormonal Signals in the Regulation of Tomato Fruit Ripening
Source: PLoS Genet. 2016 Mar 9;12(3):e1005903. doi: 10.1371/journal.pgen.1005903 (PMC4784954; doi:10.1371/journal.pgen.1005903)
Supplement: S1 Method — (PDF) [file pgen.1005903.s024.pdf]

S1 SUPPLEMENTAL METHOD

Detailed method of hormone measurements

Plant tissue was frozen under liquid nitrogen and grounded with pestle and mortar. 200 mg of frozen powder were extracted with methanol/water/formic acid (15/4/1 v/v/v) at -20°C. Internal standards were added upon the extraction. Hormones were purified and fractionated on Oasis MCX SPE cartridges (Waters). Acidic and neutral hormones were eluted with MeOH (eluate 1). Bases (cytokinins) were eluted with freshly prepared 0.35M NH_4OH in 70% MeOH (eluate 2). In order to measure cytokinin (CK) nucleotides, eluate 2 was divided to 2 equal portions: the 1st portion was injected as is, and the 2nd portion was dephosphorylated as described in (66). Concentration of CK nucleotides was calculated by subtracting corresponding ribosides concentrations in eluate 1 from eluate 2. Hormones were measured by UPLC-ESI-MS/MS equipped with Acquity UPLC H class system (Waters) and Acquity UPLC BEH C18 column (1.7um, 2.1x100mm, Waters), with gradients of 0.1% acid (acetic or formic acid) in water/acetonitrile. MS detector (Waters TQS) was equipped with ESI source. The measurement was performed in positive or negative ionization mode, using 2 MRM transitions for each compound. Quantification of compounds was done against external calibration curves, prepared by comparing the ratios of MRM peak areas of analyte to peak area of internal standard ($P_{analyte}/P_{IS}$). The same amount of internal standard was used for the preparation of both biological samples and calibration samples.
